# Peridroplet mitochondria are associated with the severity of MASLD and the prevention of MASLD by diethyldithiocarbamate

**DOI:** 10.1016/j.jlr.2024.100590

**Published:** 2024-07-07

**Authors:** Xiangyun Sun, Qinghong Yu, Yifei Qi, Bilian Kang, Xinyan Zhao, Lin Liu, Ping Wang, Min Cong, Tianhui Liu

**Affiliations:** 1 Liver Research Center, Beijing Friendship Hospital, Capital Medical University, Beijing, China; 2 Department of Hepatology, State Key Lab of Digestive Health, Beijing, China; 3 Department of Hepatology, National Clinical Research Center for Digestive Diseases, Beijing, China; 4 Beijing Key Laboratory of Translational Medicine in Liver Cirrhosis, Beijing, China

**Keywords:** lipid droplets, perilipin 5, steatotsis, proteomics, triglyceride, fatty acids

## Abstract

Mitochondria can contact lipid droplets (LDs) to form peridroplet mitochondria (PDM) which trap fatty acids in LDs by providing ATP for triglyceride synthesis and prevent lipotoxicity. However, the role of PDM in metabolic dysfunction associated steatotic liver disease (MASLD) is not clear. Here, the features of PDM in dietary MASLD models with different severity in mice were explored. Electron microscope photographs show that LDs and mitochondria rarely come into contact with each other in normal liver. In mice fed with high-fat diet, PDM can be observed in the liver as early as the beginning of steatosis in hepatocytes. For the first time, we show that PDM in mouse liver varies with the severity of MASLD. PDM and cytosolic mitochondria were isolated from the liver tissue of MASLD and analyzed by quantitative proteomics. Compared with cytosolic mitochondria, PDM have enhanced mitochondrial respiration and ATP synthesis. Diethyldithiocarbamate (DDC) alleviates choline-deficient, L-amino acid–defined diet-induced MASLD, while increases PDM in the liver. Similarly, DDC promotes the contact of mitochondria-LDs in steatotic C3A cells in vitro. Meanwhile, DDC promotes triglyceride synthesis and improves mitochondrial dysfunction in MASLD. In addition, DDC upregulates perilipin 5 both in vivo and in vitro, which is considered as a key regulator in PDM formation. Knockout of perilipin 5 inhibits the contact of mitochondria-LDs induced by DDC in C3A cells. These results demonstrate that PDM might be associated with the progression of MASLD and the prevention of MASLD by DDC.

The spectrum of metabolic dysfunction–associated steatotic liver disease (MASLD) ranges from simple steatosis to steatohepatitis (metabolic dysfunction–associated steatohepatitis, MASH), the latter is characterized with inflammation and/or fibrosis and causes high risk for cirrhosis and hepatocellular carcinoma (1). Up to now, few drugs are approved for MASH. Mitochondrial dysfunction contributes to the progression of MASH (2). Therefore, it is importance to identify potential therapeutic agents for preserving mitochondrial function for MASLD.

MASLD is defined by the abundance of lipid droplets (LDs) in hepatocytes. At the early stage for most MASLD patients, increased LDs in hepatocytes does not equate to cellular dysfunction. Hepatic LD accumulation is considered an adaptive response to the increased flow of FFAs. LDs maintain energy homeostasis and protect against lipotoxicity by sequestering toxic lipids into their core (3, 4, 5, 6). Dysregulation of LD biogenesis can increase lipid accumulation and lead to inflammation and fibrosis (7).

It has been shown that LDs can contact mitochondria and regulate the function of mitochondria in brown adipose tissue (BAT) (8). The mitochondria bound to LDs (so-called peridroplet mitochondria, PDM) have enhanced function. PDM could support LD expansion by providing ATP for triglyceride (TG) synthesis and prevent cell injury from lipotoxicity (9). Electron microscopic images of livers from high-fat diet (HFD)-induced model of obesity appear to have increased contact between mitochondria and LDs (10). Just recently, Talari’s group reported the isolation of LD-associated mitochondria from a MASLD rat model (11). However, little is known about the role of mitochondria-LDs contact or PDM in MASLD.

We previously demonstrated that diethyldithiocarbamate (DDC) attenuates methionine- and choline-deficient (MCD) diet–induced MASH in mice (12). Interestingly, steatosis increased in mice treated with DDC. The ballooning of hepatocytes was disappeared and replaced by macrovesicular steatosis, where a single large LD completely displaced the normal cytoplasm (12). Since PDM could support LD expansion and prevent cell injury from lipotoxicity, we hypothesized that the formation of PDM might be associated with the therapeutic effect of DDC on MASH.

In present study, we first explored the features of PDM in MASLD. PDM and cytoplasmic mitochondria (CM) were isolated from the liver tissues of MASLD and then analyzed by quantitative proteomics based on data-independent acquisition (DIA). In addition, the effect of DDC on PDM and the possible mechanism were investigated in vivo and in vitro.

## Materials and methods

### Animals

Six or eight-week-old male C57BL/6 mice and six-week-old male Sprague Dawley (SD) rats were purchased from Beijing HFK Bio-Technology, Beijing, China. All rodents were group-housed in a temperature-controlled room with a 12 h light-12 h dark cycle and had free access to food and water. Animal experiments were approved by the Laboratory Animal Centre, affiliated with Beijing Friendship Hospital [SYXK(Jing) 2012-0023] and were performed in accordance with institutional and national legal guidelines for animal protection. Animal studies are reported in compliance with the ARRIVE guidelines.

To establish animal models of MASLD, C57BL/6 mice were fed with MCD diet, high-fat choline-deficient L-amino acid–defined (CDAA) diet and Western diet (WD), and SD rats were fed with MCD diet, respectively. To investigate the effect of DDC on MASLD and PDM, C57BL/6 mice were fed with high-fat CDAA diet either with standard drinking water or 4 mg/ml DDC via daily drinking water. At study termination, all the fed-state rodents were killed to harvest liver tissues for different assays after anesthesia.

### Normal mice for transmission electron microscopic observation of mitochondria

Eight-week-old male C57BL/6 mice (n = 5) were fed a standard chow diet (CD, Harlan TD.7012) for 9 weeks.

### WD-induced mild steatosis in mice for transmission electron microscopic observation of mitochondria

Eight-week-old male C57BL/6 mice (n = 5) were fed ad libitum a HFD, high-carbohydrate diet (WD) with 42% kcal from fat and containing 0.1% cholesterol (Harlan TD.88137) with a high fructose-glucose solution (SW, 23.1 g/L d-fructose + 18.9 g/L d-glucose) for 2 weeks.

### WD-induced mild MASH in mice for transmission electron microscopic observation of mitochondria, isolation of PDM and CM, and DIA-based proteome profiling

Eight-week-old male C57BL/6 mice (n = 6) were fed ad libitum a HFD, high-carbohydrate diet (WD) with 42% kcal from fat and containing 0.1% cholesterol (Harlan TD.88137) with a high fructose-glucose solution (SW, 23.1 g/L d-fructose + 18.9 g/L d-glucose) for 20 weeks.

### MCD diet–induced advanced MASH in mice for transmission electron microscopic observation of mitochondria

Eight-week-old male C57BL/6 mice (n = 5) were fed MCD diet (MP Biomedicals, 960440) for 8 weeks.

### MCD diet–induced MASLD in rats for isolation of PDM and CM

Six-week-old male SD rats (n = 6) were fed MCD diet (MP Biomedicals, 960440) for 6 weeks.

### CDAA diet–induced MASH in mice for DDC treatment, isolation of PDM and CM, and transmission electron microscopic observation of mitochondria

C57BL/6 mice fed a CDAA diet show increased lipid synthesis, oxidative stress, and inflammation, resulting in liver fibrosis (13, 14). Six-week-old male C57BL/6 mice were fed with CDAA diet (Moldiets, M10462) (supplemental Table S1) to establish MASH model. After one week of acclimatization, mice were randomly assigned to the following independent groups: CD group, CDAA group and CDAA + DDC group. C57BL/6 mice were fed either a standard CD (CD, Harlan TD.7012) or CDAA diets for 9 weeks (n = 5 for CD; n = 6 for CDAA) or 15 weeks (n = 6 for CD; n = 12 for CDAA). The CDAA group were treated either with vehicle (standard drinking water) or 4 mg/ml DDC via daily drinking water for 9 weeks (n = 6 for CDAA + DDC) or 15 weeks (n = 12 for CDAA + DDC).

### Transmission electron microscopy

Liver samples (n = 5) were fixed in 2.5% glutaraldehyde in 0.1 M sodium-cacodylate buffer (pH 7.4) at 4°C for 24 h, rinsed overnight in the cacodylate buffer, following a postfixation for 12 h with 1% osmium tetroxide in cacodylate buffer. After that they were dehydrated and embedded in 618# resin. Ultrathin sections were prepared using a Reichert ultramicrotome, contrasted with uranyl acetate and lead citrate, and observed with a Philips CM120 electron microscope at 60 kV. The experiments were performed by a technician blinded to the experimental groups. For the quantification of transmission electron microscopy images, mitochondria were manually traced, and mitochondria contact with LDs was defined as PDM, while the other mitochondria were defined as CM. PDM were quantified by count, n = 10 electron micrographs per sample. For the quantification of PDM, the raw data of PDM from each sample were normalized to the number of LDs or the average value of LD perimeter.

### PDM and CM isolation from mouse liver tissues

PDM and CM were isolated by a slight modification of previously described method (15). All procedures were performed on ice with mitochondria isolation kit (Solarbio, Cat # SM0020). Seventy-five micrograms liver tissues were minced, suspended in 1 ml mitochondrial isolation buffer, and mechanically homogenized with glass homogenizer in 1.5 ml Eppendorf tube for about 20 times. After that, the homogenate was centrifuged at 1000 *g* for 5 min at 4°C to separate the fat layer containing PDM from supernatant containing CM. Then, the fat layer and supernatant were respectively transferred to new ice-cold 1.5 ml Eppendorf tube and centrifuged again with 1000 *g* at 4°C for 5 min to remove the nuclei and unbroken cells. Meanwhile, 25 μl fat layer was taken and stained in 40 μl mixture containing 1.2 μM MitoView™ Green (biotium, Cat# 70054) for mitochondria and 18 × LipidSpot™ 610 (biotium, Cat# 70069) for LDs at room temperature (RT) for 3 h, protected from light. After that, the mixture was taken to smear for confocal imaging.

Next, the pellets of CM in the supernatant and PDM in fat layer were collected with 12,000 *g* for 10 min at 4°C. To further purify the CM and PDM, the pellets were resuspended in wash buffer at 1000 *g* for 5 min at 4°C. And then, the supernatants were transferred and centrifuged again at 12,000 *g* for 10 min at 4°C to harvest the isolated CM and PDM. The purified CM and PDM were ready for the following experiments.

### Proteome profiling for PDM and CM based on DIA method

Protein was extracted from the purified CM and PDM (n = 6), and the concentration was determined using BCA method. The samples were separated by SDS-PAGE gel running, the protein distribution was observed, and the overall condition of the samples was evaluated. One hundred micrograms of protein was taken from each of the above samples, and protein digestion was performed using the filter-aided sample preparation method (16). High-performance liquid phase separation was performed by Thermo Corporation's UltiMate 3000 UHPLC, and then the peptides separated by the liquid phase were separated and ionized by a nanoESI source and then transferred to a tandem mass spectrometer Q-Exactive HF (Thermo Fisher Scientific, San Jose, CA) for DIA mode detection. The proteomic analysis software Proteome Discoverer 2.1 was used to perform protein identification analysis on the data collected by DDA, and the results of identification are used for DIA quantitative protein. The search database was downloaded by UniProt website. The search results adopted a screening condition of false discovery rate ≤1% at the peptide level and a screening condition of Q value ≤1% at the protein level. The proteins are then quantified. Briefly, the quantification values are peak areas for precursor ion. Then, normalization method is used to remove systematic deviation in the peptide intensity values between LC-MS measurements. As for the normalization method, the percentage of abundance of each protein in each sample is calculated according to the results of protein quantification, and then multiplied by the average of the abundance sum of each sample; the normalized results of protein quantification can be obtained. Differential proteins were mapped into biological processes or pathways using Gene Ontology and Kyoto Encyclopedia of Genes and Genomes. For further details regarding the proteomic analysis, please refer to the supplementary information.

### RNAscope in situ hybridization

Formalin-fixed, paraffin embedded liver sections were used for RNAscope in situ hybridization as we previously reported (17). Target retrieval (Advanced Cell Diagnostics [ACD], REF 322000) was performed for 30 min. The probes used were against positive control (*Mm-Ppib*) (ACD, REF 313911), negative control (*Mm-Dap-B*) (ACD, REF 310043), perilipin 5 (*Mm-Plin5*) (ACD, REF 865111). Chromogenic Assays were carried out using the RNAscope® 2.5 HD Detection Reagent-RED (ACD, REF 322360) as recommended by the manufacturer (ACD, https://www.acdbio.com/technical-support/user-manuals. Cat. No. 322452-USM and Cat. No. 322360-USM). In particular, the liver tissue slides were incubated for 40 min at RT in AMP5 stage.

### Histochemistry and immunohistochemical analyses

As we previously reported (12), H&E was performed for determination of steatosis and lobular inflammation (18). Collagen was stained as we previously reported (19), too. Immunohistochemistry (IHC) was performed as we previously reported (12). Quantification in the average of five randomly selected fields per section (20 × magnification) (n = 6) for IHC staining of cluster of differentiation 68–positive macrophages. The antibodies used in IHC were listed in supplemental Table S2.

### Western blotting

Protein was extracted from liver tissues or cells through RIPA buffer lysis (Solarbio, Cat# R0010), and the sample protein concentrations were determined using the BCA protein assay (Thermo Fisher scientific, REF 23227). After incubating with the primary antibody, the signal was amplified either by an anti-mouse (ZSGB-BIO, Beijing, ZB-2305) or anti-rabbit (ZSGB-BIO, Beijing, ZB-2301) HRP-conjugated secondary antibody. All values were normalized to β-actin expression. Antibodies were listed in supplemental Table S2.

### RNA extraction and quantitative real-time PCR

The total RNA was extracted with Trizol® Reagent (Ambion, REF 15596018) from liver tissues followed by reverse-transcription (TOYOBO, Cat# FSQ-301). Quantitative real-time PCR (qPCR) was performed with a 7500 real-time PCR system (Applied Biosystems) using SYBR green chemistry (Applied Biosystems, REF A46109). qPCR was performed with 40 cycles of 5 s at 95°C and 30 s at 60°C after a 2-min initial enzyme activation at 95°C. Expression of *β-actin* was used to standardize the samples, and the results were expressed as a ratio relative to control. Relative expression was calculated using 2 ^(-ΔΔCT)^. All experiments were carried out at least three times independently, and the averages were used for the comparisons. The primer sequences used for qPCR were listed in supplemental Table S3.

### Lipidomic analyses of mouse liver specimens

Liver specimens were thawed on ice (n = 5 for each group). Take 50 mg of one sample and homogenize it with 1 ml mixture (include methanol, methyl tert-butyl ether and internal standard mixture) and steel ball. As for the composition of the 1 ml mixture, the internal standard mixture (listed in supplemental Table S4) was dissolved in the buffer including methyl tert-butyl ether and methanol at a ratio of 5:1. Take out the steel ball and whirl the mixture for 15 min. Add 200 μl of water and whirl the mixture for 1 min, and then centrifuge it with 12,000 rpm at 4°C for 10 min. Extract 300 μl supernatant and concentrate it. The powder was dissolved for LC-MS/MS analysis. The UHPLC separation was carried out using a 1290 Infinity series UHPLC System (Agilent Technologies), equipped with a Kinetex C18 column. The Triple TOF mass spectrometer was used for its ability to acquire MS/MS spectra on an information-dependent basis during an LC/MS experiment.

An in-house program, namely, LipidAnalyzer, was developed using R for automatic data analysis. The raw data files (.wiff format) were converted to files in mzXML format using the “msconvert” program from ProteoWizard (version 3.0.6150). Then, the mzxML files were loaded into LipidAnalyzer for data processing. Peak detection was first applied to the MS1 data. The CentWave algorithm in various forms (X) of chromatography mass spectrometry was used for peak detection, with the MS/MS spectrum, lipid identification was achieved through a spectral match using an in-house MS/MS spectral library. The absolute quantitation of lipids were achieved using the peak area, stable isotope-labeled internal and response factor information. The internal lipid standards contain heavy isotopes and they can be distinguished from endogenous lipids. When quantifying lipid projects, these internal standards were used to evaluate recovery rates when developing methods. During detection, the data were normalized using internal standards and then obtain accurate content based on the standard curve.

### Detection of ATP content in mitochondria

The concentration of ATP in PDM or CM was measured with a commercial assay kit according to the manufacturer's instructions (BC0305, Beijing Solarbio Science & Technology Co., Ltd China).

### Detection of malondialdehyde in the liver tissues

The concentration of malondialdehyde (MDA) in the liver tissues was measured with a commercial assay kit according to the manufacturer's instructions (S0131S, Beijing Solarbio Science & Technology Co., Ltd China).

### Induction of steatosis and DDC treatment for C3A cells

The hepatocytic cell line, C3A cell (ATCC® CRL-10741), a subclone of the human hepatoma cell line (HepG2), was cultured as we previously reported (20, 21). 0.5 mM FFAs (oleic acid (Sigma-Aldrich, Cat# O1257): palmitic acid (Sigma-Aldrich, Cat# P0500)= 1 : 10) were obtained as previously described method (19, 22, 23). The C3A cells were exposed to complete medium containing 0.5 mM FFAs for 24 h. After exposure, the steatotic C3A cells treated with or without 100 μM DDC for 24 h. All cells were harvested for different assays.

### RNA interference

*PLIN5* siRNA and negative control siRNA were purchased from Ambion (Cat # 4392422). The C3A cells were transfected with 30 nM of specific or negative control siRNA using riboFECT ™ CP transfection Kit (Ribobio, China, Cat # C10511-05). When the cells were 24 h post-transfection, they were ready for the following experiments.

### Staining of mitochondria and LDs in C3A cells

One hundred nano meter MitoView™ Green was used for mitochondria staining at RT for 30 min, and 1 × Hoechst 33,342 (Beyotime, Cat# C1028) were added to stain the nuclei for 10 min. As for LD staining, C3A cells were rinsed and incubated with complete medium containing 1 × LipidSpot™ 610 at RT for 30 min, protected from light as we previously reported (19). After that, a laser scanning confocal microscope (Olympus Fluoview FV1000) was used to capture 6 different fields per well (n = 5). For the quantification of confocal images, the amount of LDs was assessed as the area of LipidSpot™ 610. Mitochondria-LDs contact was assessed as the area of mitochondria colocalized with LD. n ≥60 C3A cells analyzed per group from five independent experiments.

### Statistical analysis

Data were carried out by GraphPad Prism software (version 9. 1. 0), and all data are expressed as mean ± SD. Statistical analysis was performed using the Student’s *t* test for comparing two selected groups, paired *t* test for comparing PDM and CM, and one-way ANOVA for comparing multiple groups. The criterion for statistical significance was defined at *P* value <0.05.

## Results

### PDM appear in the liver at the early stage of MASLD and varied with the severity of MASLD

In steatotic liver, abundant LDs exist in hepatocytes and are similar with adipocyte morphologically. In addition, the features of PDM in BAT have a strong resemblance to the changes of mitochondrial function when hepatic lipogenesis increased (24). It might have been speculated that the mitochondria-LDs contact (PDM) play a role in MASLD. To test this, dietary MASLD models with different severity were established in mice.

**Fig. 1:**
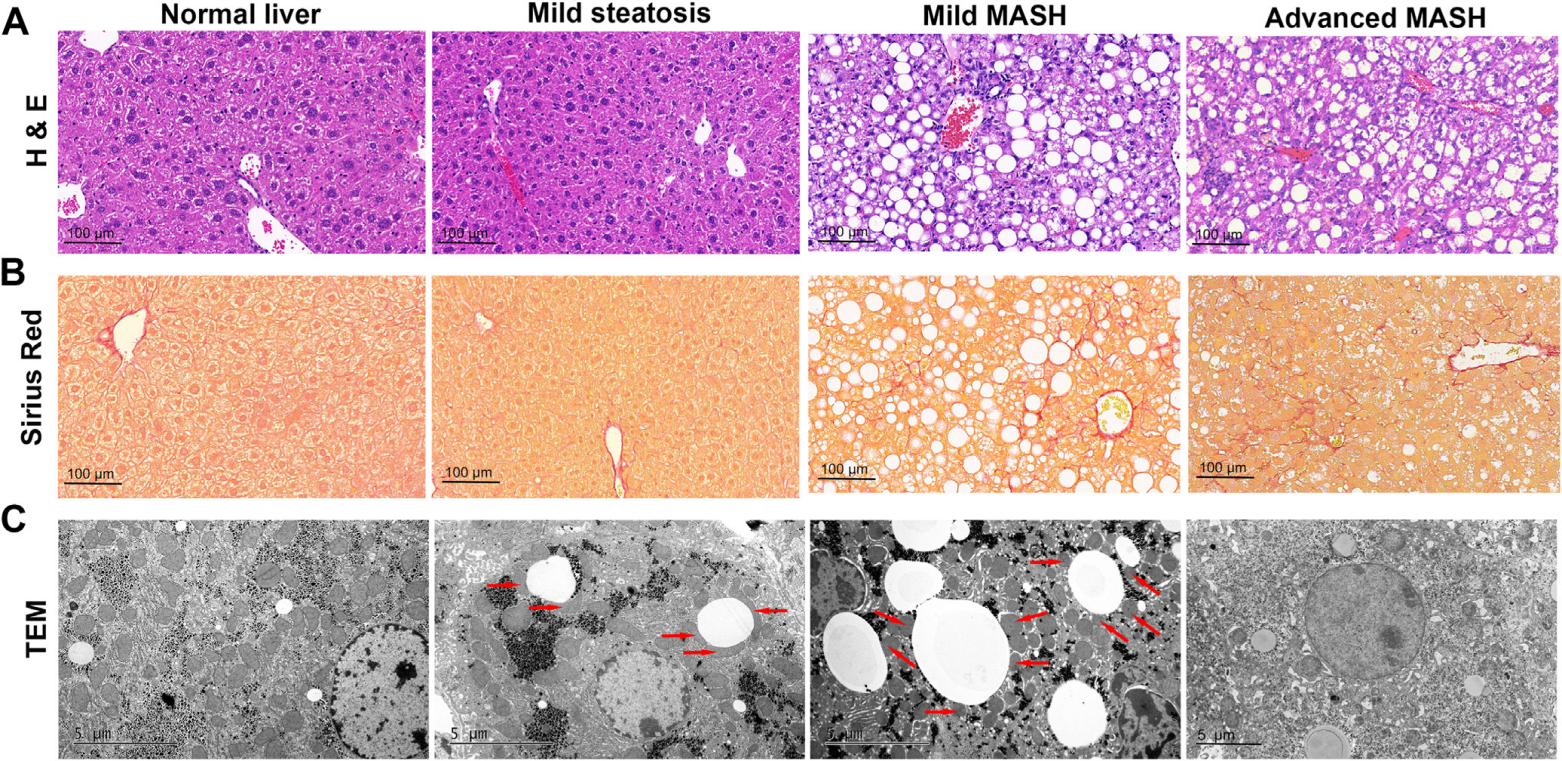
PDM in mouse liver tissues with different severity of MASLD. Dietary MASLD models with different severity have been established. The normal mice were fed a chow diet (CD) for 9 weeks, the mice in mild steatosis were fed a WD diet for 2 weeks, the mice in mild MASH were fed a WD diet for 20 weeks, and the mice in advanced MASH were fed MCD diet for 8 weeks. (A) Representative H&E stainings of mouse liver tissues with different severity of MASLD (n = 5). Scale bars, 100 μm. (B) Representative Sirius Red stainings of mouse liver tissues with different severity of MASLD (n = 5). Scale bars, 100 μm. (C)Transmission electron micrographs of mouse liver tissues with different severity of MASLD (n = 5). Scale bars, 5 μm. Red arrows point to mitochondria which contact with LDs (PDM). MCD, methionine and choline deficient; MASH, metabolic dysfunction–associated steatohepatitis; MASLD, metabolic dysfunction–associated steatotic liver disease; PDM, peridroplet mitochondria; WD, Western diet.

In healthy liver tissues, the electron microscope photographs showed that there are only few and small LDs in hepatocytes. In addition, LDs and mitochondria usually exist independently and rarely come into contact with each other (Fig. 1). By the time the mice were fed a WD diet for 2 weeks, the hepatocytes containing LDs are rarely seen in H&E stainings and no perisinusoidal fibrosis. Since there are insufficient steatosis for a diagnosis of MASLD (a threshold of 5% of hepatocytes showing steatosis) (25, 26), we called this histologic changes as mild steatosis. However, electron microscopy showed that distinct LDs were visible in some hepatocytes compared to healthy tissue, and the LDs always contact with mitochondria and form PDM in the liver tissue of mild steatosis (Fig. 1). Some LDs are even completely surrounded by mitochondria (supplemental Fig. S1). In the liver tissues of MASH mice with mild perisinusoidal fibrosis, electron microscopy revealed obvious LDs in nearly all hepatocytes. The number of PDM increased compared to mice with mild steatosis (supplemental Fig. S2). However, some LDs are surrounded by only a few mitochondria, and LDs surrounded by mitochondria are rare. In advanced MASH with severe ballooning of hepatocytes and pericellular fibrosis, LDs decrease and PDM are hardly seen in hepatocytes (Fig. 1 and supplemental Fig. S2). These data indicated that PDM begin to appear in the liver at the early stage of MASLD, and the number of PDM varies according to the severity of MASLD.

### PDM have different characteristics from CM in steatotic liver

**Fig. 2:**
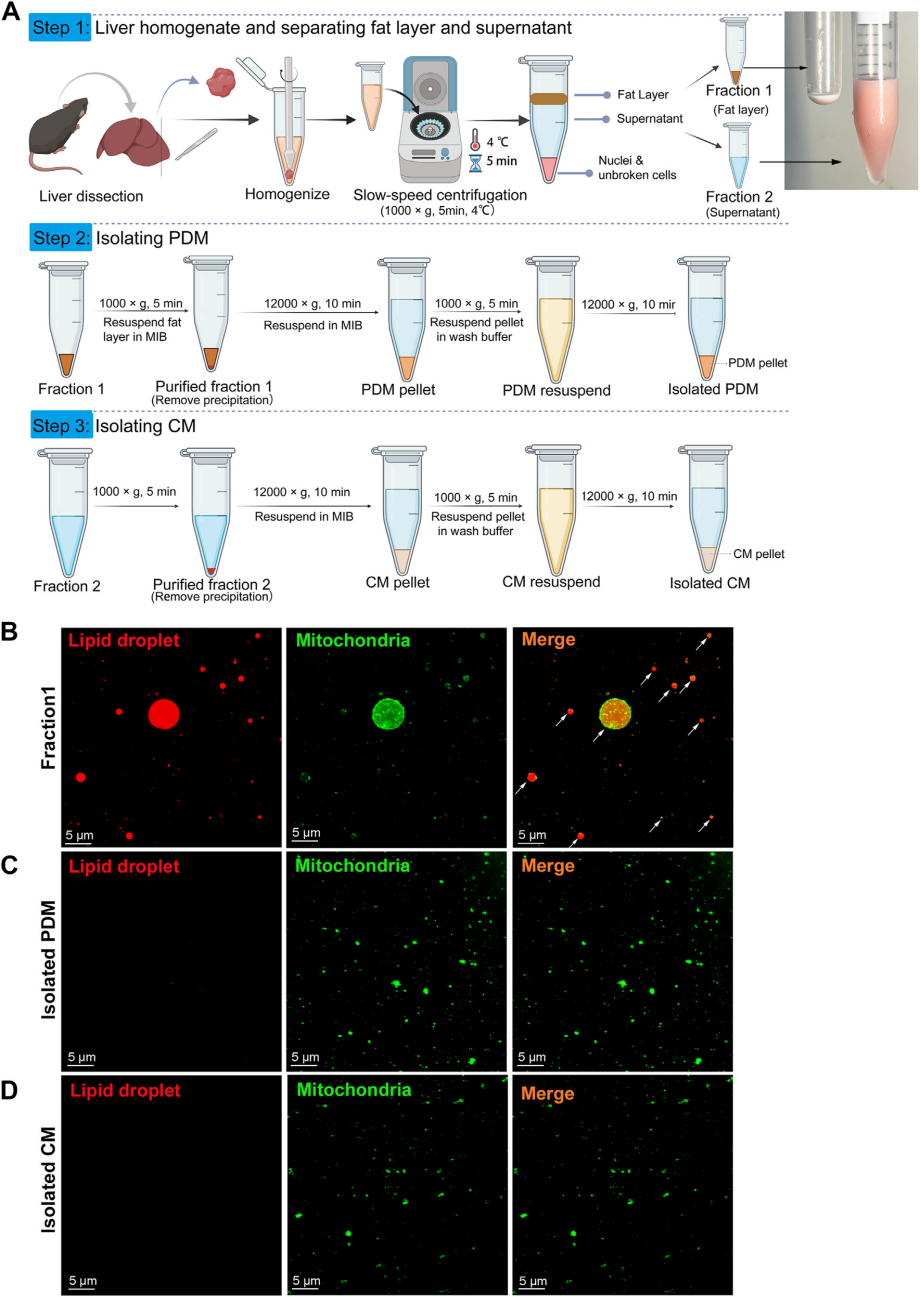
Isolation of PDM and CM in steatotic liver of MASLD. (A) Schematic representation of PDM and CM isolation procedure. Eight-week-old male C57BL/6 mice were fed a WD diet for 20 weeks. The liver tissues were dissected from mice and homogenized. Low-speed centrifugation separated the fat layer containing PDM from supernatant containing CM. High-speed centrifugation stripped PDM from LDs and pelleted CM from the supernatant. (B) Confocal image of PDM in fraction 1 (fat layer) before high-speed centrifugation. Twenty-five microliters fat layer were taken and costained with MitoView™ Green (green) for mitochondria and LipidSpot™ 610 (red) for LDs (n = 5). White arrows point to the LDs surrounded by mitochondria (PDM). Scale bar, 5 μm. (C) Confocal images of isolated PDM. PDM were isolated from fat layer by high-speed centrifugation and were costained with MitoView™ Green (green) and LipidSpot™ 610 (red) (n = 5). Scale bar, 5 μm. (D) Confocal images of isolated CM. CM were isolated from fraction 2 (supernatant) by high-speed centrifugation and were costained with MitoView™ Green (green) and LipidSpot ™ 610 (red) (n = 5). Scale bar, 5 μm. CM, cytoplasmic mitochondria; LD, lipid droplet; PDM, peridroplet mitochondria; WD, Western diet.

To further investigate the features of PDM in MASLD, the mitochondria were isolated from the steatotic liver based on their adherence to LDs by differential centrifugation. Here, the WD diet–induced MASLD mice for 20 weeks were used. As shown in Figure 2A, the liver tissues were harvested and homogenized by dounce homogenizer. After low-speed centrifugation, the mitochondria contacted with LDs (PDM) will locate in the upper fat layer and the mitochondria not contacted with LDs (CM) will locate in the supernatant under the fat layer.

To demonstrate the existence of PDM, the fat layer was costained for mitochondria and LDs. As shown in Figure 2B, confocal microscopy revealed that some LDs (Red) were surrounded by mitochondria (Green)-PDM in the fat layer. Next, to obtain the purified mitochondria, the mitochondria located in the fat layer and the supernatant were centrifuged by high-speed centrifugation, respectively. After high-speed centrifugation, LDs will be stripped out from PDM. The isolated PDM pellet was costained for mitochondria and LDs. As shown in Figure 2C, confocal microscopy revealed that little to no LipidSpot ™ 610 staining, suggesting that PDM were successfully separated from LDs. As shown in supplemental Fig. S3, the stripped fat layer were stained with LipidSpot™ 610 and numerous LDs can be seen, suggesting that the majority of PDM were harvested from LDs. As shown in Figure 2D, the isolated CM were costained with MitoView™ Green and LipidSpot™ 610, and little to no LipidSpot™ 610 staining can be seen, suggesting that CM are devoid of associated LDs.

To compare the difference between PDM and CM, proteome profiling based on DIA was used. The staining of Coomassie brilliant blue showed that the distribution pattern of proteins in PDM and CM are significantly different (supplemental Fig. S4A). More than 1500 proteins were detected in isolated PDM and CM. However, almost all proteins were detected in both PDM and CM, and no specific proteins for PDM or CM were found. One sample of CM was removed because of less than 600 proteins were identified. Two hundred sixty differentially expressed proteins were identified. Among these proteins, 199 proteins are upregulated and 61 proteins are downregulated in PDM (supplemental Fig. S4B). Differentially expressed proteins were mapped into biological processes and cellular components or pathways using Kyoto Encyclopedia of Genes and Genomes (supplemental Fig. S4C) and Gene Ontology (supplemental Fig. S4D). The PDM proteome has a higher number of oxidative phosphorylation (OXPHOS), fatty acid biosynthesis, cholesterol metabolism, ABC transporters, and thermogenesis-related proteins showing higher expressions, while CM proteome has a higher number of fatty acid β-oxidation, fatty acid degradation, and peroxisome-related proteins showing higher expressions (supplemental Fig. S4C, D). Overall, through proteomic study, the data suggest that the PDM are functionally different from CM in steatotic liver tissues of MASLD.

### PDM have higher levels of OXPHOS and ATP in steatotic liver of MASLD

**Fig. 3:**
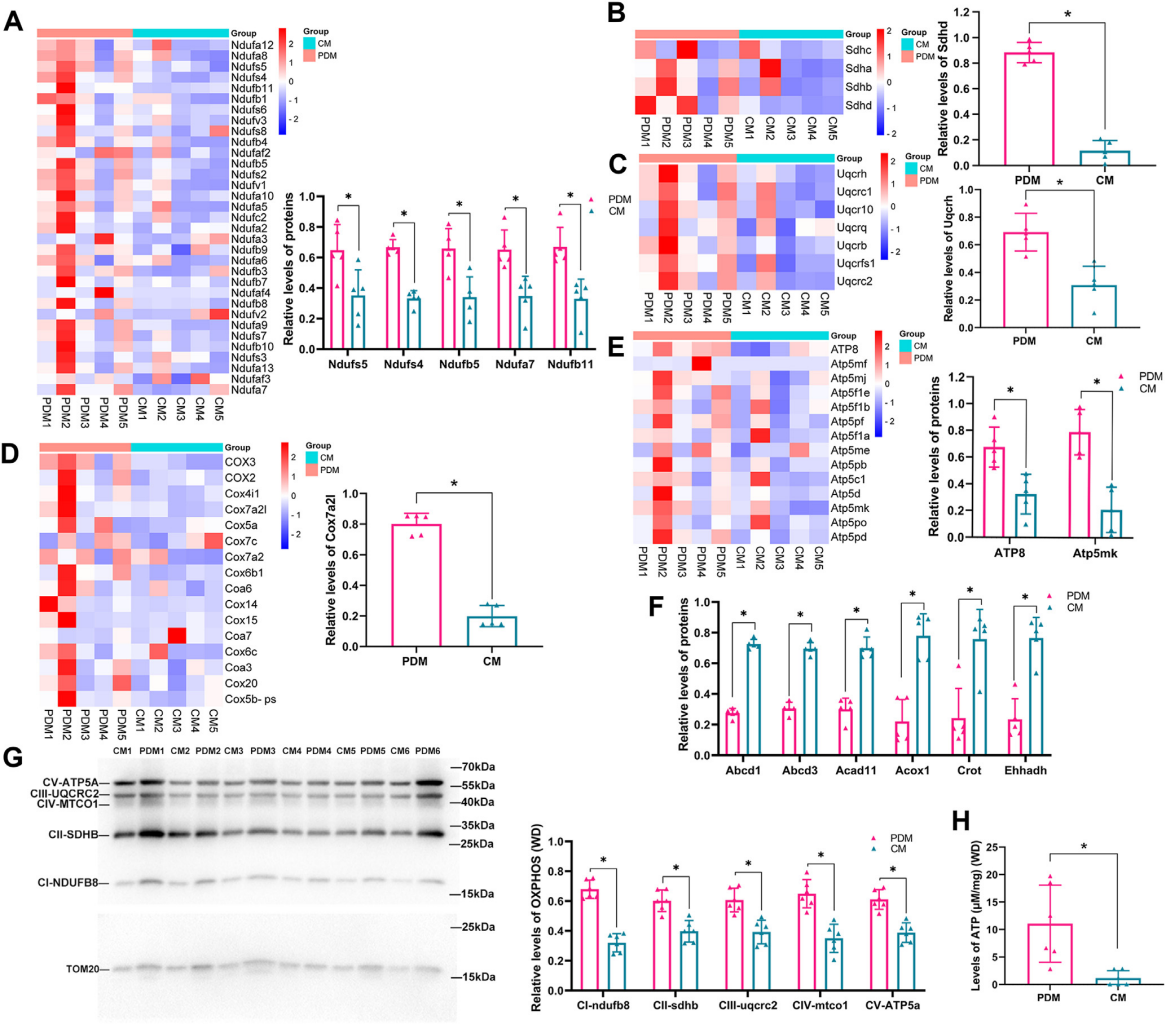
The difference of OXPHOS complex and fatty acid β-oxidation–related proteins between PDM and CM in steatotic liver of MASLD. Eight-week-old male C57BL/6 mice were fed a WD diet for 20 weeks (A–E) Heat map and the higher levels of OXPHOS complex I–V–related proteins in PDM versus CM (n = 5). (F) Relative levels of fatty acid β-oxidation–related proteins in PDM versus CM (n = 5). (G) Western blot of antibodies of OXPHOS complex I–V in PDM and CM isolated from 20-week WD–induced steatotic liver of MASLD mice, and TOM20 was used as a loading control (n = 6). The semiquantification of OXPHOS complex I–V in PDM and CM isolated from 20-weeks WD-induced steatotic liver of MASLD mice (n = 6). First quantification of OXPHOS complex subunits were normalized to TOM20 loading control to obtain the raw data. For independent samples, the raw data of CM and PDM from each individual sample were normalized to the sum of PDM and CM in that sample as shown in the formula: PDM_normalized_ = PDM_raw_/PDM_raw_ + CM_raw_, CM_normalized_ = CM_raw_/PDM_raw_ + CM_raw_. (H) Relative levels of ATP content in PDM and CM isolated from 20-week WD–induced steatotic liver of MASLD mice (n = 6). The data are expressed as the mean ± SD and analysed by paired *t* test for comparing PDM and CM. ∗*P* < 0.05, denotes differences between the compared group. CM, cytoplasmic mitochondria; OXPHOS, oxidative phosphorylation; MASLD, metabolic dysfunction–associated steatotic liver disease; PDM, peridroplet mitochondria; WD, Western diet.

The OXPHOS system is central to the mitochondrial function. The relative abundance of OXPHOS complex related proteins were then analyzed, and the accession numbers of proteins are listed in supplemental Table S5. As shown in Figure 3A, more than 30 proteins related to complex I (NADH: CoQ oxidoreductase enzyme) were detected, including the structural proteins and assembly factors. PDM have higher levels of NADH dehydrogenase iron-sulfur protein 4 and 5 (Ndufs4, Ndufs5), NADH dehydrogenase 1 beta subcomplex subunit 5 and 11 (Ndufb5, Ndufb11), and NADH dehydrogenase 1 alpha subcomplex subunit 5 (Ndufa7). As shown in Figure 3B, four structural proteins of complex II (succinate dehydrogenase) were detected. Mitochondrial succinate dehydrogenase is the only enzyme complex engaged in both OXPHOS and the tricarboxylic acid cycle. It is one of the key enzymes reflecting mitochondrial function and can also reflect the capacity of tricarboxylic (27). PDM have higher levels of succinate dehydrogenase cytochrome b small subunit protein relative to CM. As shown in Figure 3C, seven structural proteins of complex III (cytochrome bc1 oxidoreductase) were detected. PDM have higher levels of cytochrome b-c1 complex subunit 6 (Uqcrh) protein relative to CM. As shown in Figure 3D, 16 structural proteins and assembly factors of complex IV (cytochrome c oxidase) were detected. PDM have higher levels of cytochrome c oxidase subunit 7A–related protein (Cox7A2l) relative to CM. As shown in Figure 3E, 14 structural proteins of complex V (ATP synthase) were detected. PDM have higher levels of ATP synthase protein 8 and ATP synthase membrane subunit K protein relative to CM.

Benador *et al.* reported that PDM in BAT have reduced fatty acid oxidation capacity. The relative abundance of fatty acid β-oxidation related proteins were then analyzed, and the accession numbers of proteins are listed in supplemental Table S5. As shown in Figure 3F, six differentially expressed proteins related to fatty acid β-oxidation were identified. All of these proteins are lower in PDM relative to CM, including acyl-CoA dehydrogenase family member 11, ABC subfamily D member , ABC subfamily D member 3, carnitine O-octanoyltransferase, acyl-coenzyme A oxidase 1, and peroxisomal L-bifunctional enzyme (Ehhadh).

To further validate the difference of OXPHOS between PDM and CM, PDM and CM were isolated from the liver tissues in different rodent MASLD models: WD diet-induced MASLD in mice, CDAA diet–induced MASLD in mice, and MCD diet–induced MASLD in rats. In all the three kinds of MASLD models, Western blot analysis revealed that the OXPHOS complex I–V levels in PDM were higher than those in CM (Fig. 3G and supplemental Fig. S5C, D), and the OXPHOS complex I–Ⅲ, Ⅴ levels in PDM were higher than those in CM (supplemental Fig. S5A, B). In addition, the concentration of ATP in PDM and CM were detected in WD diet–induced MASLD mice. As shown in Figure 3H, PDM has higher levels of ATP than CM. Collectively, these data suggest that the PDM have enhanced mitochondrial respiration and ATP synthesis than CM.

### DDC attenuates CDAA diet–induced MASH in mice and promotes the formation of PDM in vivo

Since PDM have enhanced mitochondrial respiration and ATP synthesis, it is reasonable to speculate that PDM play a positive role in MASLD through preserving the function of mitochondria. Here, we tried to investigate whether PDM could be regulated in vivo by DDC, which we previously demonstrated its protective effect on MCD diet–induced MASH in mice (12).

**Fig. 4:**
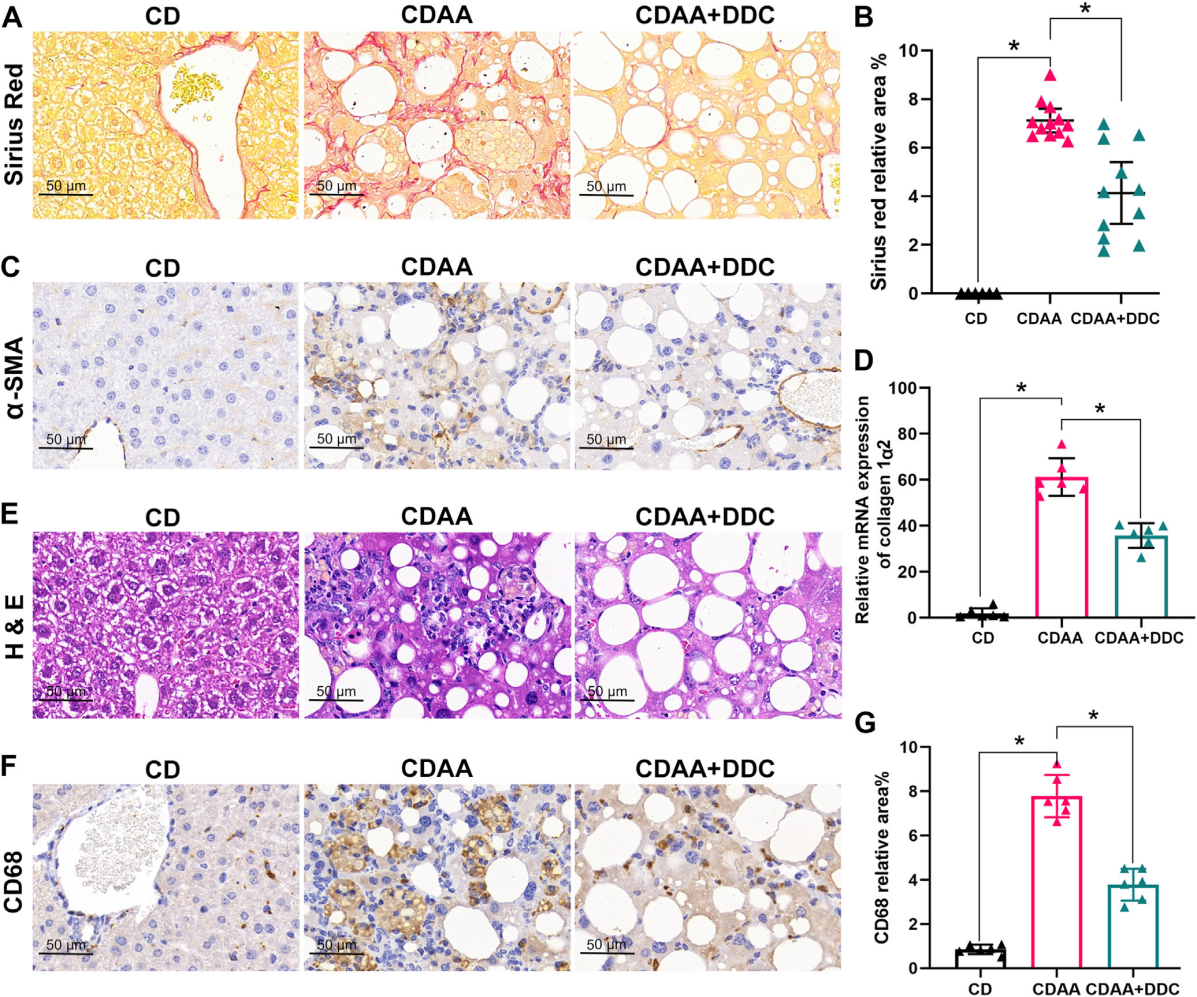
DDC alleviates hepatic fibrosis and inflammation in CDAA diet-induced MASH mice. Six-week-old male C57BL/6 mice were fed a CD diet or a CDAA diet for 15 weeks. The CDAA group were treated either with standard drinking water or 4 mg/ml DDC via daily drinking water. (A) Representative image of Sirius Red staining, scale bar, 50 μm. (B) The quantification of Sirius Red area (n = 6 for CD, n = 12 for CDAA, n = 11 for CDAA + DDC). (C) Representative image of IHC staining of α-SMA (n = 6), scale bar, 50 μm. (D) Relative mRNA levels of *Collagen 1α2* in liver tissues (n = 6). (E) Representative image of H&E staining, scale bar, 50 μm. (F, G) Representative image of IHC staining of CD68 and quantification in the average of five randomly selected fields per section (20× magnification) (n = 6), scale bar, 50 μm. Data are expressed as mean ± SD and analyzed by one-way ANOVA with multiple comparisons and Tukey post hoc test. ∗*P* < 0.05, denotes differences between the compared group. CD, chow diet; CD68, cluster of differentiation 68; CDAA, choline-deficient l-amino acid–defined; CM, cytoplasmic mitochondria; DDC, diethyldithiocarbamate; IHC, immunohistochemistry; MASH, metabolic dysfunction–associated steatohepatitis; α-SMA, α-smooth muscle actin.

First, the therapeutic effect of DDC on CDAA diet–induced MASH was validated. One sample was removed from the CDAA + DDC group because of death. Compared with CD group, mice on CDAA diet developed pericellular fibrosis (Fig. 4A and supplemental Fig. S6A), while the collagen disposition significantly decreased in DDC-treated group (Fig. 4B and supplemental Fig. S6B). Equally, α-smooth muscle actin expression increased in CDAA group and decreased in DDC-treated group (Fig. 4C and supplemental Fig. S6C). Accordingly, the elevated mRNA of *Collagen 1α2* in CDAA group was suppressed by DDC treatment (Fig. 4D and supplemental Fig. S6D). In addition, the lobular inflammation and cluster of differentiation 68+ macrophages increased in CDAA group, while DDC inhibited these increases (Fig. 4E–G and supplemental Fig. S6E, F). Oxidative stress and Oxidative stress–induced lipid peroxidation are involved in the pathogenesis of MASLD. It is defined as an imbalance between the production and elimination of reactive oxygen species (ROS). ROS can oxidize macromolecules, resulting in the formation of toxic products. For example, lipid peroxidation results in the formation of products such as MDA, which is considered a biomarker for oxidative damage of lipids and can reflect the levels of ROS in the liver indirectly (28). As shown in supplemental Fig. S7, compared with the normal control, the level of MDA in the liver increased significantly in CDAA diet–induced MASLD group, while decreased in DDC-treated group. These data indicated that ROS was reduced in DDC treated mouse livers.

**Fig. 5:**
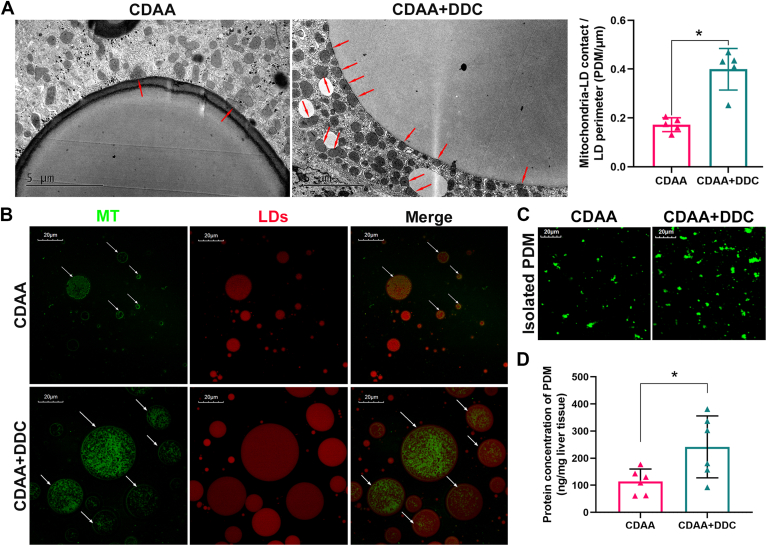
DDC promotes the formation of PDM in vivo. Six-week-old male C57BL/6 mice were fed a CD diet or a CDAA diet for 9 or 15 weeks. The CDAA group was treated either with standard drinking water or 4 mg/ml DDC via daily drinking water. (A) Representative image of electron micrographs of liver tissues from 9-week CDAA diet–induced MASH mice without or with DDC treatment showing mitochondria and LDs (n = 5). Scale bar, 5 μm. Red arrows point to mitochondria which contact with LD (PDM). Mitochondria were manually traced, and mitochondria contact with LD were defined as PDM, while the other mitochondria were defined as CM. PDM were quantified by count. n = 10 electron micrographs per sample. The raw data of PDM from each sample were normalized to the average value of LD perimeter. (B and C) Liver tissues of 15-week CDAA diet–induced MASH mice was homogenized and centrifugated at 1000 *g* to generate a floating fat layer containing PDM and a supernatant containing CM. Twenty-five microliters fat layer were taken and co-stained with MitoView™ Green (green) for mitochondria and LipidSpot™ 610 (red) for LDs (n = 5) in the panel B. White arrows point to the LDs surrounded by mitochondria (PDM). Then, PDM were isolated from fat layer by high-speed centrifugation at 12,000 *g*, and were stained with MitoView™ Green (green). Confocal images of isolated PDM (n = 5) in the panel C. Scale bar, 20 μm. (D) Relative quantification of PDM yield in liver tissue from 9-week CDAA diet–induced MASH mice and DDC-treated mice (n = 6). The data are expressed as the mean ± SD and analyzed by the Student’s *t* test for comparing two selected groups. ∗*P* < 0.05, denotes differences between the compared group. CD, chow diet; CM, cytoplasmic mitochondria; CDAA, choline-deficient l-amino acid–defined; DDC, diethyldithiocarbamate; LD, lipid droplet; MASH, metabolic dysfunction–associated steatohepatitis; PDM, peridroplet mitochondria.

Compared with the CDAA diet–induced MASH group, a large amount of mitochondria was recruited to the surface of LDs (PDM) after DDC treatment (Fig. 5A). To further demonstrate the effect of DDC of PDM formation, the fat layer of steatotic liver tissues was co-stained for mitochondria and LDs. As shown in Figure 5B, confocal microscopy revealed that some LDs (Red) were surrounded by mitochondria (green)-PDM in the fat layer. There are more large LDs and PDM in DDC-treated group than those in MASH group. Accordingly, the results of fluorescent staining show that the purified PDM extracted from fat layer by high-speed centrifugation were markedly increased by DDC treatment (Fig. 5C). Moreover, to further quantify the PDM, the yield obtained after PDM purification was estimated. As shown in Figure 5D, the content of PDM protein obtained from the same amount of liver tissue was significantly higher in DDC-treated mice. Collectively, these data indicate that DDC promotes the formation of PDM.

### DDC promotes TG synthesis in the liver of CDAA diet–induced MASH mice and improves the function of mitochondria

**Fig. 6:**
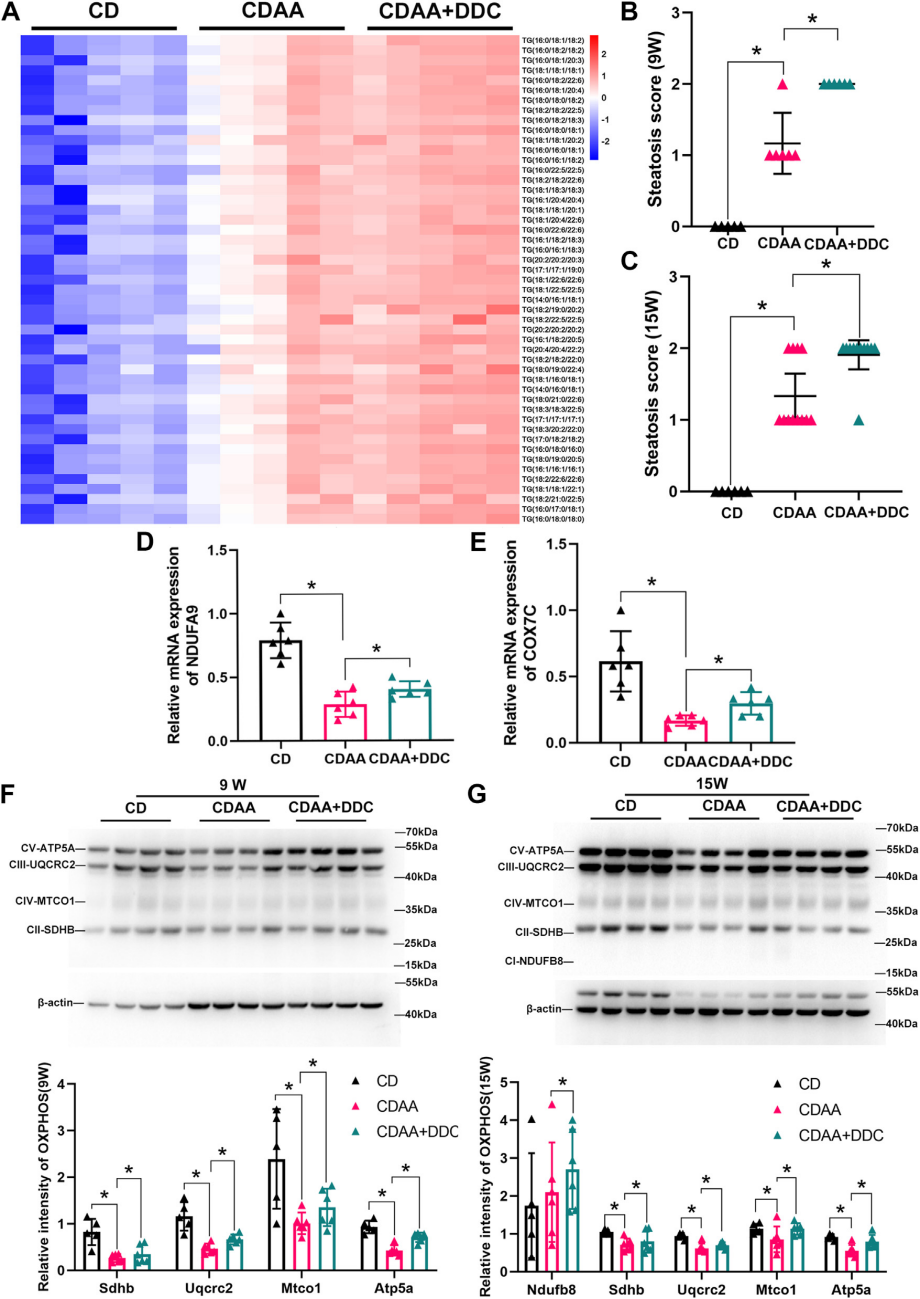
DDC promotes TG synthesis and enhances mitochondrial respiration in the liver of CDAA diet–induced MASH mice. Male C57BL/6 mice were fed either CD or CDAA diets for 9 or 15 weeks, respectively. The CDAA group were treated either with standard drinking water or 4 mg/ml DDC via daily drinking water. (A) Liver tissue homogenates were harvested from 9-week CDAA diet–induced MASH mice for lipidomic analyses. Heatmap of different types of TGs expression from the indicated groups (n = 5 liver specimens per group). (B) Hepatic steatosis score for all mice in the average of 10 randomly selected fields per section of H&E staining (20 × magnification) in mice fed either CD or CDAA diets for 9 weeks (n = 5 for CD; n = 6 for CDAA; n = 6 for CDAA + DDC). (C) Hepatic steatosis score for all mice in the average of ten randomly selected fields per section of H&E staining (20× magnification) in mice fed either CD or CDAA diets for 15 weeks (n = 6 for CD; n = 12 for CDAA; n = 11 for CDAA + DDC). (D, E) Relative mRNA levels of *Ndufa9* and *C*o*x7c* in liver tissues of 15-week CDAA diet–induced MASH mice (n = 6). (F) The total protein was extracted from the liver tissues. The OXPHOS complex II–V were detected by Western blot in the livers of 9-week mice (n = 6). Quantification of OXPHOS complex subunits normalized to β-actin loading control (G) The total protein was extracted from the liver tissues. The OXPHOS complex I–V were detected by Western blot in the livers of 15-week mice (n = 6). Quantification of OXPHOS complex subunits normalized to β-actin loading control data are expressed as mean ± SD and analyzed by one-way ANOVA with multiple comparisons and Tukey post hoc test. ∗*P* < 0.05, denotes differences between the compared group. CD, chow diet; CDAA, choline-deficient l-amino acid–defined; DDC, diethyldithiocarbamate; MASH, metabolic dysfunction–associated steatohepatitis; OXPHOS, oxidative phosphorylation; TG, triglyceride.

It has been reported that PDM could support LD expansion by providing ATP for TG synthesis and prevent cell injury from lipotoxicity (9). Since DDC promotes the formation of PDM, the content of TG in the liver tissues was then analyzed by lipidomic analyses. The data showed that the contents of 206 types of hepatic TG increased in DDC-treated group compared with CDAA diet group (Fig. 6A and supplemental Table S6). Accordingly, though CDAA diet–induced inflammation and fibrosis were significantly attenuated, steatosis was not improved but even increased by DDC (Fig. 6B, C). Meanwhile, the ratio of liver weight to body weight increased in DDC-treated group (supplemental Fig. S8).

Since PDM have higher levels of OXPHOS complex proteins, the question then was whether DDC could improve the dysfunction of mitochondria. The mRNA of NADH dehydrogenase (ubiquinone) 1 alpha subcomplex, 9 (*Ndufa9*) and cytochrome C oxidase 7c (*Cox7c*) decreased in the liver of CDAA group, while it is increased upon DDC-treated group (Fig. 6D, E). Compared with the CD group, OXPHOS complex II–V levels decreased in the liver of mice fed CDAA diets for both 9 weeks and 15 weeks, indicating the dysfunction of mitochondria in MASH (Fig. 6F, G). Compared with the CDAA group, OXPHOS complex II–V levels increased in the liver of mice fed CDAA diets for 9 weeks upon DDC treatment (Fig. 6F), and OXPHOS complex I–V levels increased in the liver of mice fed CDAA diets for 15 weeks upon DDC treatment (Fig. 6G), indicating that DDC improves the dysfunction of mitochondria.

### Plin5 is upregulated by DDC in the liver of CDAA diet–induced MASH mice

**Fig. 7:**
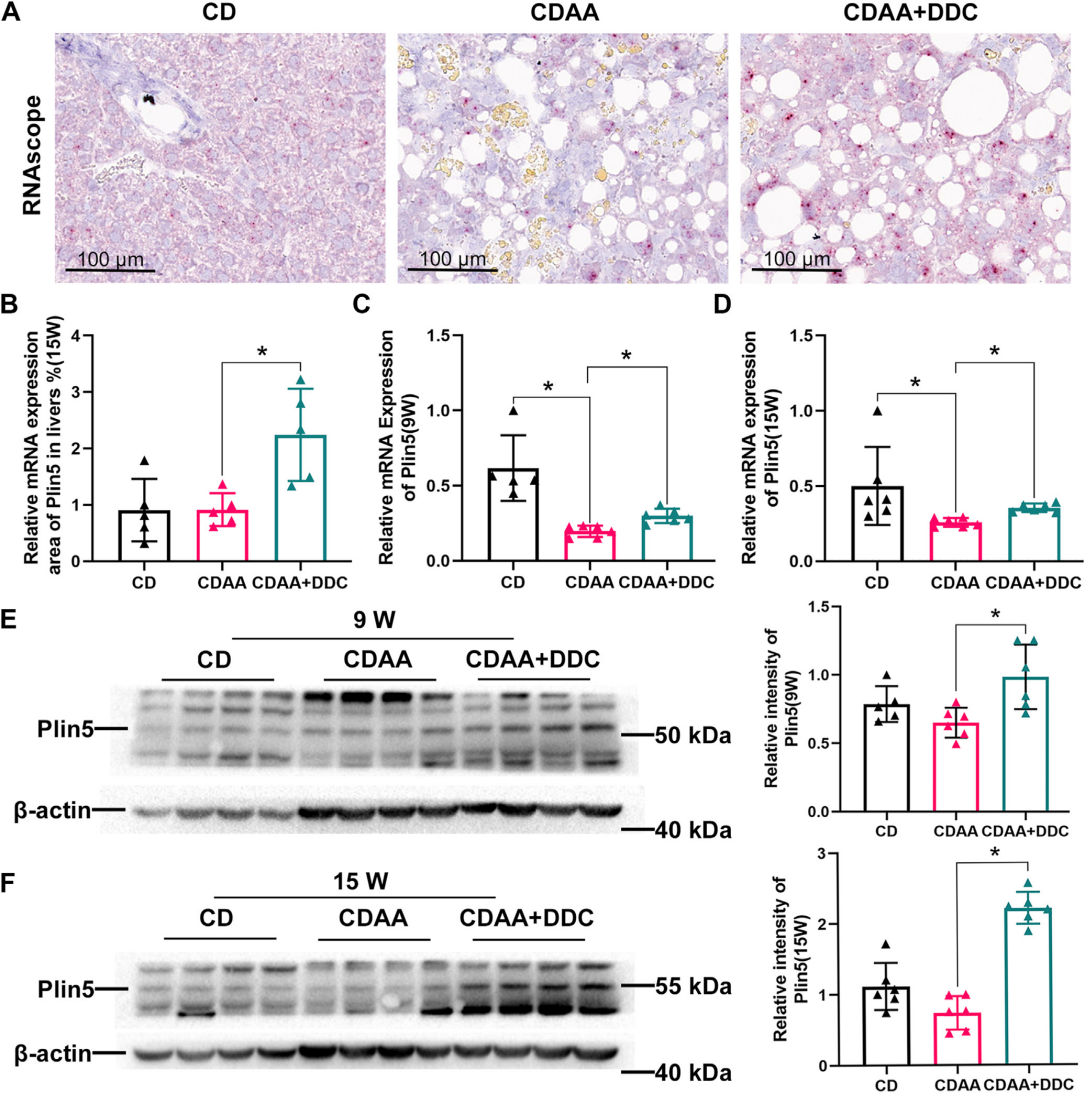
Plin5 is upregulated by DDC in the liver of CDAA diet-induced MASH mice. Male C57BL/6 mice were fed either CD or CDAA diets for 9 or 15 weeks, respectively. (A, B) RNAscope of chromogenic assays for *Plin5* (red) and quantification of *Plin5* in livers of 15-week mice (n = 6). Scale bars, 100 μm. (C, D) Relative mRNA levels of *Plin5* detected by qPCR in livers of 9 and 15-week mice (n = 6). (E, F) Western blot analysis and relative density ratio of Plin5 to β-actin in livers of 9 and 15-week mice (n = 6). All data represent the mean ± SD. One-way ANOVA with Tukey post test. ∗*P* < 0.05. MASH, metabolic dysfunction–associated steatohepatitis; Plin5, perilipin 5; qPCR, quantitative real-time PCR; DDC, diethyldithiocarbamate; CD, chow diet; CDAA, choline-deficient l-amino acid–defined.

It has been reported that Plin5 could recruit mitochondria to the surface of LDs and form the PDM in BAT (9). It has been investigated whether DDC promotes the PDM formation through regulating Plin5. First, the *Plin5* mRNA distribution was detected by chromogenic RNAscope in situ in the liver of CDAA diet-induced MASH mice. Compared with the CDAA mice, RNAscope displayed significantly higher *Plin5* mRNA (red)-positive region in DDC-treated group (Fig. 7A, B). Accordingly, RT-PCR results showed that *Plin5* mRNA was increased in the liver tissues by DDC treatment when compared with CDAA group (Fig. 7C, D). Furthermore, protein levels of Plin5 were increased by DDC treatment (Fig. 7E, F). These data uniformly show that DDC upregulates Plin5 in the liver of CDAA diet–induced MASH mice.

### DDC promotes the contact of mitochondria-LDs through upregulating PLIN5 in vitro

**Fig. 8:**
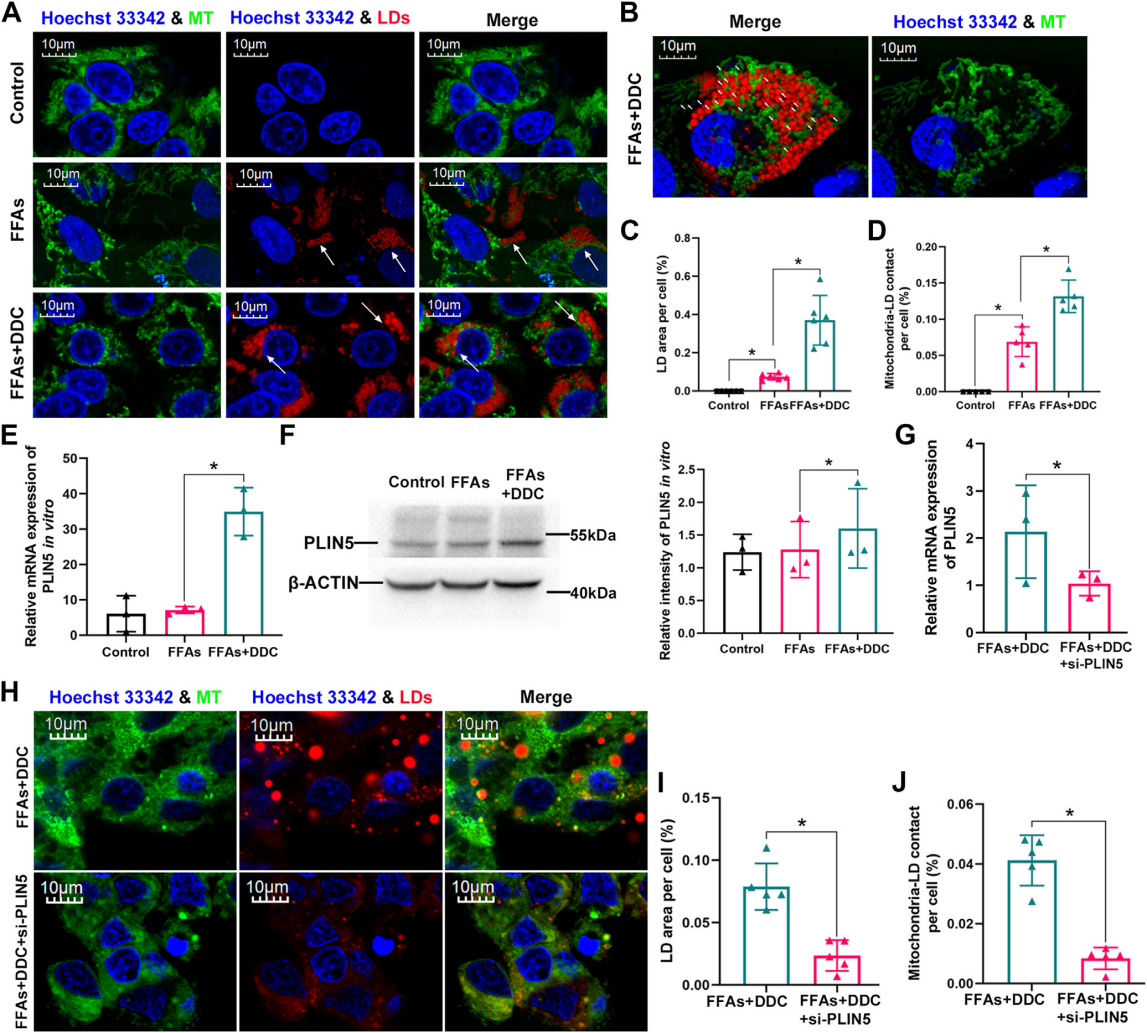
DDC promotes the contact of mitochondria-LDs through regulating PLIN5 in vitro. Mixture (OA: PA = 1: 10) were used to treat C3A cells without (FFAs) or with DDC (FFAs + DDC). (A) The C3A cells were stained with the LipidSpot™ 610 and MitoView™ Green and observed by confocal microscope. White arrows point to LDs. Scale bar, 10 μm. (n = 5). (B) Super-resolution confocal image staining of one cell in FFAs + DDC group. White arrows point to the contact of mitochondria-LDs. Scale bar, 10 μm. (C, D) The amount of LDs was assessed as the area of LipidSpot™ 610. And mitochondria-LD contact was assessed as the area of mitochondria colocalized with LDs. n ≥ 60 C3A cells analyzed per group from five independent experiments. (E) Relative mRNA levels of *PLIN5* in C3A cells. The expression of target gene was normalized to that of *β-ACTIN*. These experiments were repeated at least three times. (n ≥ 3). (F) Western blot analysis of PLIN5 in C3A cells and relative density ratio of PLIN5 to β-ACTIN. These experiments were repeated at least three times. (n ≥ 3). (G) si-*PLIN5* was transfected to silence the expression of *PLIN5* in DDC-treated steatotic C3A cells. Relative mRNA levels of P*LIN5* in C3A cells were detected by qPCR. These experiments were repeated at least three times. (n ≥ 3). (H) The C3A cells were stained with the LipidSpot™ 610 and MitoView™ Green and observed by confocal microscope. Scale bar, 10 μm. (n = 5). (I, J) The amount of LDs was assessed as the area of LipidSpot™ 610. And mitochondria-LDs contact was assessed as the area of mitochondria colocalized with LDs. n ≥60 C3A cells analyzed per group from five independent experiments. Hoechst 33,342 was used to visualize nuclei (blue) in the panels A, B, and H. All data represent the mean ± SD. Student’s *t* test for comparing two selected groups and one-way ANOVA with Tukey post test was used to compare multiple groups. ∗*P* < 0.05. DDC, diethyldithiocarbamate; PLIN5, perilipin 5; qPCR, quantitative real-time PCR; LD, lipid droplet; OA, oleic acid; LD, lipid droplet; PA, palmitic acid.

To further investigate the impact of DDC on the contact of mitochondria-LDs (PDM) in vitro, the steatosis was induced by FFAs in C3A cells, a subclone of the human hepatoma cell line (HepG2). As shown in Figure 8A, B, compared with FFAs-treated group, the content of LDs were significantly increased in DDC-treated cells (Fig. 8C). Furthermore, the LDs were more closely linked to the mitochondria by DDC treatment (Fig. 8D). These data further demonstrated that DDC promotes LD formation and the contact of mitochondria-LDs.

To further validated the regulation of DDC on PLIN5, steatosis was induced by culturing C3A cells in 0.5 mM FFAs. After inducing steatosis for 24 h, DDC was administered on the steatotic C3A cells for 24 h. Then, the C3A cells were collected to examine the mRNA and protein levels of PLIN5. As shown in Fig. 8E, the expression of *PLIN5* mRNA was significantly increased by DDC treatment. Accordingly, PLIN5 protein expression significantly upregulated upon treatment with 100 μM DDC relative to DDC-untreated steatotic C3A cells (Fig. 8F). These data were consistent with the upregulation of PLIN5 in steatotic hepatocytes by DDC in vivo.

To investigate whether PLIN5 is required for the contact of mitochondria-LDs in this context, we performed the experiment of *PLIN5* knockdown in FFAs + DDC–treated C3A cells (Fig. 8G). As shown in the Figure 8H, compared with FFAs + DDC–treated group, the content of LDs was significantly decreased after *PLIN5* was knockdown (Fig. 8I). In addition, the contact of mitochondria-LDs decreased after *PLIN5* was knockdown (Fig. 8J). These data indicated that DDC promotes LD formation and the contact of mitochondria-LDs which might be associated with the upregulation of PLIN5 by DDC.

## Discussion

Mitochondria are dynamic and form contacts with other cellular organelles. Interorganelle contacts can impact the function of mitochondria, and the alterations of mitochondria-organelle contacts might lead to the metabolic dysfunction in steatotic liver diseases (29, 30). In this study, the features of mitochondria which contact with LDs (PDM) were investigated in steatotic liver of MASLD mice. The data show that PDM in the liver have enhanced mitochondrial respiration and ATP synthesis. In addition, the number of PDM in the liver varies according to the severity of MASLD. For the first time, we provide the evidence that the formation of PDM can be regulated in vivo and are associated with the therapeutic effect of DDC on MASLD.

PDM was first discovered and named in BAT, and there is also some evidence for the contact between mitochondria and LDs in the liver of rodents with obesity and MASLD (9, 10, 11). In this study, PDM were observed in MASLD liver tissues with different severity in mice using transmission electron microscopy. The results show that PDM is rarely present in normal liver tissue. Interestingly, when mice were fed HFD, PDM began to appear in the liver of the mice at a very early stage, even before obvious steatosis was present (mild steatosis). Since PDM could trap the dietary excess of fatty acids in LDs, the appearance of PDM might be a compensatory response to excess nutrition at this time. At the early stage of MASH, PDM can also be observed. However, PDM are hardly seen in the advanced MASH with severe ballooning of hepatocytes and pericellular fibrosis. These data indicate that PDM might be associated with the progression of MASLD.

Recently, Ilan Benador’s group developed an approach to isolate PDM from BAT based on their adherence to LDs. Compared with CM, PDM in BAT have enhanced pyruvate oxidation and ATP synthesis capacities, and reduced β-oxidation capacity (9). In this study, PDM and CM were isolated from steatotic liver of MASLD mice and analyzed the difference between PDM and CM by proteome profiling based on DIA. The proteomic study reveals that the PDM are functionally different from CM in steatotic liver tissues of MASLD. Similar with the PDM in BAT, PDM in steatotic liver tissues of MASLD have higher levels of proteins related to mitochondrial respiration and ATP synthesis and lower levels of proteins related to fatty acid β-oxidation. However, Talari *et al.* reported that LD-associated mitochondria in liver tissues of MASLD Wistar rats exhibit higher fatty acid oxidation and are marked by enhanced levels of MFN2 and carnitine palmitoyltransferase, while CM are associated with higher respiration capacity (11). To further validate the features of PDM in MASLD, PDM and CM were isolated from the MASLD liver in both C57/BL6 mice and SD rats. The results reveals that the OXPHOS complex levels in PDM are higher than those in CM in all these MASLD models. Since PDM have increased respiratory and ATP synthesis capacities, it is reasonable to speculate that PDM might play a positive role in preventing the progression of MASLD.

We then investigated whether PDM could be regulated by DDC which we previously demonstrated its protective function on MASH (12). In this study, DDC significantly attenuates CDAA diet–induced hepatic inflammation and liver fibrosis in mice. However, DDC treatment increases the degree of steatosis in mice fed with CDAA diet. In addition, hepatic TG increases in DDC-treated mice, which is exactly consistent with the increase of steatosis. It has been demonstrated that PDM could support LD expansion by providing ATP for TG synthesis and enable the cells to perform antagonistic metabolic processes (9). The formation of PDM may be an event of mitochondrial adaptation to overcome the increased import of FFAs. Consistent with that, the amount of PDM in steatotic liver of MASLD is increased in DDC-treated mice, which might explain the increase of hepatic TG and steatosis after DDC treatment. The in vitro data also prove that DDC promotes the contact of mitochondria-LDs accompanying with increased LDs. These data indicate that PDM might be involved in the prevention of MASH by DDC. Accordingly, we conclude that DDC promotes the formation of PDM accompanied with enhanced mitochondrial function, which might be associated with the improvement of CDAA diet–induced MASH.

Plin5 is a LD protein which regulates the contents of TG in hepatocytes and protects against oxidative stress (31). In addition, overexpression of Plin5 increases LD mass and protects against lipotoxic liver injury in mice (32, 33). In Benador’s study, it has been shown that mitochondria-LDs contact is highly regulated by Plin5 in cardiac myocytes and brown adipocytes (9). Wang *et al.* reported that mitochondria-LDs contact is regulated by Plin5 in cardiac myocytes (34). In Gallardo's study, they observed a significant change in the content of LDs after knockdown or overexpression of Plin5. However, knockdown of Plin5 did not change the extent of LD interactions with mitochondria in brown adipocytes. As for Plin5 overexpression, the mitochondria in contact with LDs in brown adipocytes decreases at 23°C, but increases at 6°C (35). Additionally, the overexpression of Plin5 promotes LD formation and mitochondria-LDs contact and enhances mitochondrial function in HepG2 cells (31). Our data show that Plin5 significantly increases in mouse liver and in steatotic hepatocytes when treated with DDC, while the content of LDs and the contact of mitochondria-LDs decreased after Plin5 was knockdown. These data indicate that DDC might promote the formation of PDM through upregulating Plin5 and then improve mitochondrial dysfunction in MASH.

In summary, at the early stage of MASLD (simple steatosis) and even before the formation of obvious steatosis, to overcome the increased import of FFAs in liver, mitochondria are recruited to LDs to form PDM. PDM support LD expansion by providing ATP for TG synthesis, and protect against fatty acid “spillover” and lipotoxic stress in hepatocytes. At the late stage of MASLD (advanced MASH), the number of PDM decrease. The ability of mitochondria to overcome increased FFAs is insufficient. Along with the continuously external fatty acid overload, increased lipotoxicity leads to mitochondrial dysfunction, hepatic injury, and subsequent inflammation and fibrosis. DDC promotes the formation of PDM, which in turn preserves the mitochondrial function, and inhibits the progression of MASH (supplemental Fig. S9).

However, the present study still has some limitations. In this study, we used the protocol reported by Ngo *et al.* to isolate PDM and CM with some modification (36). The possible reason is that interorganelle contacts are ubiquitous, and every organelle forms functional contacts with atleast one other organelle and often with more than one (37). It is demonstrated that mitochondria can contact with ER, LD, peroxisome, lysosome, and Golgi apparatus (38), that might explain the possible contamination of other organelles and cellular compartments. The present protocol cannot completely solve the problem of extraction purity, and it is necessary to optimize the protocol in the future. Despite lots of data available on mitochondria-LDs contact, the molecular regulation of mitochondria-LDs contact remains largely unknown. We only investigated the effect of DDC on Plin5 that has already well-studied in the regulation of PDM. The mechanisms by which DDC affects PDM should be further elucidated. In addition, human investigations that confirm findings in mice are needed in future studies. In conclusion, results presented here report that PDM with enhanced mitochondrial function and ATP synthesis exist in steatotic liver of MASLD and may be associated with the progression of MASLD. Plin5 regulated PDM are associated with the prevention of MASLD by DDC. Our findings suggest that regulation of PDM may represent a new pharmacological strategy for MASLD.

## Data availability

The data that support the findings of this study are available from the corresponding author upon reasonable request. The mass spectrometry proteomics data have been deposited to the ProteomeXchange Consortium (http://proteomecentral.proteomexchange.org) via the iProX partner repository (39, 40) with the dataset identifier PXD047208.

## Supplemental data

This article contains supplemental data (41).

## Conflicts of interest

The authors declare that they have no conflicts of interest with the contents of this article.
