# Supplementary material for: Peridroplet mitochondria are associated with the severity of MASLD and the prevention of MASLD by diethyldithiocarbamate
Source: J Lipid Res. 2024 Jul 7;65(8):100590. doi: 10.1016/j.jlr.2024.100590 (PMC11381863; doi:10.1016/j.jlr.2024.100590)
Supplement: Supporting information [file mmc1.docx]

**Title:** Peridroplet mitochondria are associated with the severity of MASLD and the prevention of MASLD by diethyldithiocarbamate

**Contents**

1. Supplementary Material and Methods
2. Supplementary Figures
3. Supplementary Tables

**Supplementary Material and Methods**

**Proteome profiling for PDM and CM based on DIA method**

Protein was extracted from the purified CM and PDM, and was analyzed by proteome profiling based on DIA method. The protocols are as followed in detail.

**1 Sample collection and processing**

**Protein extraction**

1) Add 6 mL protein extraction buffer to the sample (500 mM Tris-HCl buffer, 50 mM

EDTA, 700 mM sucrose, 100 mM KCl solution, 2% β-mercaptoethanol and 1 mM

benzylsulfonyl fluoride, adjust the pH to 8.0 with HCl), Mix evenly and grind the liquid

nitrogen for 10 minutes;

2) Add equal volume of Tris saturated phenol and fully grind for 10 min;

3) Transfer the solution to a 50 mL pre-cooled centrifuge tube, 5500 g, and centrifuge at

4 ℃ for 10 min;

4) Pipette the protein-containing phenol solution into a new pre-cooled centrifuge tube;

5) Add 6 mL of extraction buffer, 5500 g, and centrifuge at 4 ℃ for 10 min;

6) Transfer the phenol phase to a new centrifuge tube, add 4 times the volume of

pre-cooled acetone, and precipitate overnight at -20 ℃;

7) 5500 g, 4 ℃, centrifuge for 10 min;

8) Discard the supernatant and add 2 ml of pre-cooled acetone;

9) 5500 g, 4 ℃, centrifuge for 10 min;

10) Discard the supernatant and dry in a precipitation fume hood;

11) Add 4% SDS to dissolve the protein pellet.

**Protein quantification and detection**

1) Protein concentration determination using BCA or Bradford method.

2) The samples were separated by SDS-PAGE gel running, the protein distribution was

observed, and the overall condition of the samples was evaluated.

**FASP digestion**

100 µg of protein was taken from each of the above samples and protein digestion was

performed using the FASP method, specific steps are as follows:

1) Add 8 M Urea to each sample to make up to 200ul;

2) Add DTT to each sample to a final concentration of 10 mM and react at 37 ° C for 2.5 h;

3) Add IAA to each sample to a final concentration of 50 mM, and react at room temperature in the dark for 40 minutes;

4) Transfer each sample to a 10 K ultrafiltration tube;

5) 12000 g, centrifuge at room temperature, discard the filtrate;

6) Add 400 ul 8 M Urea 12000 g, centrifuge at room temperature, discard the filtrate, and

repeat twice;

7) Add 400 ul of 50 mM ammonium bicarbonate solution to the ultrafiltration tube, 12000

g, centrifuge at room temperature, discard the filtrate, and repeat 3 times;

8) Add 200ul of 50 mM ammonium bicarbonate solution to the ultrafiltration tube;

9) Add trypsin to the ultrafiltration tube, the ratio is sample / enzyme = 50/1 (mass ratio),

and hydrolyze at 37 ° C for 16 h;

10) Centrifuge at 12000 g at 4 ° C, collect the filtrate, and freeze-dry.

**High pH RP classification**

Each sample taken after the enzymatic portion of the polypeptide, using High pH RP

grading,

1) Add 1ml methanol to activate the column;

2) Add 1ml 80% acetonitrile to clean the column;

3) Add 1 ml of pH 10 ammonia balance column;

4) Reconstitute the above peptides with 200 uL of ammonia at pH 10;

5) Load the sample onto the chromatographic column;

6) Add 1ml ammonia pH 10 to clean the column;

7) Elution was carried out with 10 different gradient concentrations of acetonitrile (6%，

9%，12%，15%，18%，21%，25%，30%，35%，50%) in pH 10 ammonia solution;

8) The above ten components were combined into 3 components and lyophilized.

**2 High-performance liquid separation and DIA mass detection**

High-performance liquid phase separation was performed by Thermo Corporation's UltiMate 3000 UHPLC, and then the peptides separated by the liquid phase were separated and ionized by a nanoESI source and then transferred to a tandem mass spectrometer Q-Exactive HF (Thermo Fisher Scientific, San Jose, CA) for DIA (data-independent acquisition) mode detection.

**3 Protein identification**

Construction of Protein DDA Quantitative Database

First, the proteomic analysis software Proteome Discoverer 2.1 was used to perform protein

identification analysis on the data collected by DDA, Identification results for DIA

quantitative protein use.and the results of identification are used for DIA quantitative protein.

The search database uses the database downloaded by Uniprot website. The search engine

uses Sequest HT,.At the same time, in order to remove false positive results, the data

retrieval uses a positive and negative database retrieval strategy. The search results adopted a

screening condition of FDR ≤ 1% at the peptide level and a screening condition of Q Value ≤

1% at the protein level.

**4 Protein quantification**

Using Skyline software to build the data collected by DDA into a quantitative database,

then compare the collected DIA raw data with this quantitative database. Extract the

quantitative chromatographic peak area of the unique peptide of each protein. Using this as a

characterization of the relative abundance of a protein. The protein abundance in each sample

was compared to screen differentially expressed proteins.

**5 Differential protein screening**

We use mapDIA software for protein differential expression analysis. MapDIA is a protein

differential analysis software based on Bayesian model for DIA technology. It can find

differential proteins more accurately than other simple statistical tests.

**6 Differential protein function enrichment analysis**

GO (Gene Ontology) is an internationally standardized classification system for gene

function descriptions. GO is divided into three major types of ontology: Biological Process,

Molecular Function, and Cellular Component, which are used to describe the biological

processes involved in the products encoded by genes, the molecular functions and The

cellular environment in which it is located. The basic unit of GO is term, and each term has a

unique identifier (composed of "GO:" plus 7 numbers, such as GO: 0072669); The term of

each type of ontology constitutes a directed acyclic topology through the connection between them (is_a, part_of, regulate).

KEGG (Kyoto Encyclopedia of Genes and Genomes) is a database that systematically

analyzes the metabolic pathways of gene products and compounds in cells and the functions

of these gene products. It integrates data on the genome, chemical molecules and

biochemical systems, including metabolic pathways (KEGG PATHWAY), drugs (KEGG

DRUG), diseases (KEGG DISEASE), functional models (KEGG MODULE), gene

sequences (KEGG GENES) and genome (KEGG GENOME) and so on. The KO (KEGG

ORTHOLOG) system links various KEGG annotation systems. KEGG has established a

complete KO annotation system that can complete the functional annotation of the genome

or transcriptome of newly sequenced species. KEGG enrichment analysis is helpful to further

understand the biological function and significance of proteins, and to determine the most

important biochemical metabolic pathways and signaling pathways involved in proteins. In

organisms, different genes coordinate their biological functions, and the significant

enrichment of Pathway can determine the most important biochemical metabolic pathways

and signal transduction pathways involved in differentially expressed genes. KEGG (Kyoto

Encyclopedia of Genes and Genomes) is the main public database on Pathway (Kanehisa,

2008). Pathway significant enrichment analysis uses KEGG Pathway as a unit and applies a

hypergeometric test to find Pathway that is significantly enriched in differentially expressed

genes compared to the entire genome background. We used KOBAS (2.0) for Pathway enrichment analysis.

**Supplementary Figures**

**Supplementary Figure 1** Electron micrographs of LDs which are surrounded by mitochondria in mouse liver tissues with mild steatosis. Scale bars, 2 μm. Red arrows point to the LDs which are surrounded by mitochondria.

**Supplementary Figure 2 The quantification of PDM in the liver tissues with different severity of MASLD.** Mitochondria were manually traced, and mitochondria contact with LDs were defined as PDM while the other mitochondria were defined as CM. PDM were quantified by count. n = 10 EMs per sample. The raw data of PDM from each sample were normalized to the number of LDs. n = 5 for each group. All data represent the mean ± SD. One-way ANOVA with Tukey post-test. *p < 0.05.

**Supplementary Figure 3** LDs separated from PDM were stained with LipidSpot^TM^ 610. Scale bars, 10 µm or 5 µm indicated in the figures.

**Supplementary Figure 4 Proteome profiling of PDM and CM in steatotic liver of MASLD.**

Male C57BL/6 mice were fed WD for 20 weeks. PDM and CM were isolated from the liver tissues. (A) The staining of Coomassie brilliant blue showing the distribution pattern of proteins in PDM and CM (n=6). (B) Differentially expressed proteins identified in PDM vs CM (n=5). (C) The enriched KEGG pathways related proteins showing higher expressions in PDM vs CM (n=5). (D) The enriched GO biological process, cellular component and molecular function related proteins showing lower expressions in PDM vs CM (n=5).

**Supplementary Figure 5 The difference of OXPHOS complex between PDM and CM in steatotic liver of MASLD.**

(A) Western blot of antibodies of OXPHOS complex I-Ⅲ, Ⅴ in PDM and CM isolated from 9-week CDAA diet-induced steatotic liver of MASLD mice, and TOM20 was used as a loading control (n=6). (B) Relative levels of OXPHOS complex I-Ⅲ, Ⅴ in PDM and CM isolated from 9-week CDAA diet-induced steatotic liver of MASLD mice (n=6). (C) Western blot of antibodies of OXPHOS complex I-V in PDM and CM isolated from 6-week MCD diet-induced steatotic liver of MASLD rats (n=6). TOM20 is used as a loading control. (D) Relative levels of OXPHOS complex I-V in PDM and CM isolated from 6-week MCD diet-induced steatotic liver of MASLD rats. First quantification of OXPHOS complex subunits were normalized to TOM20 loading control to obtain the raw data. For independent samples, the raw data of CM and PDM from each individual sample were normalized to the sum of PDM and CM in that sample as shown in the formula: PDM_normalized_ = PDM_raw_/PDM_raw_ + CM_raw_, CM_normalized_ = CM_raw_/PDM_raw_ + CM_raw_. The data are expressed as the mean ± SD and analysed by paired t-test for comparing PDM and CM. *p<0.05, denotes differences between the compared group.

**Supplementary Figure 6 DDC attenuates CDAA diet-induced MASH in mice.**

Male C57BL/6 mice were fed either CD or CDAA diets for 9 weeks. The CDAA group were treated either with vehicle (standard drinking water) or 4 mg/ml DDC via daily drinking water. (A-B) Representative image of Sirius Red staining and the quantification of Sirius Red area. (C) Representative image of IHC staining of α-SMA. (D) Relative mRNA levels of *Collagen 1α2* in liver tissues. (E) Representative image of H&E staining. (F) Representative image of IHC staining of CD68. n = 5 for CD, n = 6 for CDAA, n = 6 for CDAA + DDC. Scale bars, 50 μm. All data represent the mean ± SD. One-way ANOVA with Tukey post-test. *p < 0.05.

**Supplementary Figure 7** Male C57BL/6 mice were fed either CD or CDAA diets for 15 weeks. The CDAA group were treated either with vehicle (standard drinking water) or 4 mg/ml DDC via daily drinking water. The concentration of MDA in the liver tissues was measured (n = 6). All data represent the mean ± SD. One-way ANOVA with Tukey post-test. *p < 0.05.

**Supplementary Figure 8** Male C57BL/6 mice were fed either CD or CDAA diets for 15 weeks. The CDAA group were treated either with vehicle (standard drinking water) or 4 mg/ml DDC via daily drinking water. (A) Body weight of mice. (B) Liver weight of mice. (C) Liver/body weight ratio of mice. n = 6 for CD; n = 11 for CDAA; n = 9 for CDAA + DDC. All data represent the mean ± SD. One-way ANOVA with Tukey post-test. *p < 0.05.

**Supplementary Figure 9** The features of PDM in steatotic liver of MASLD and the possible therapeutic mechanism of DDC through modulating PDM formation.

**Supplementary table 1. Formula of CDAA diet (M10462S)**

| Ingredient | gm | |
| --- | --- | --- |
| Casein | 0.0 | |
| Corn Starch | 0.0 | |
| Maltodextrin | 130.1 | |
| Sucrose | 68.8 | |
| Cellulose BW200 | 50.0 | |
| Soybean Oil | 25.0 | |
| Lard | 245.0 | |
| L-Cystine | 4.2 | |
| L- Isoleucine | 7.6 | |
| L-Leucine | 15.8 | |
| L-Lysine | 13.2 | |
| L-Methionine | 0.8 | |
| L-Phenylalanine | 8.4 | |
| L-Threonine | 7.2 | |
| L-Tryptophan | 2.1 | |
| L-Valine | 9.3 | |
| L-Histidine | 4.6 | |
| L-Alanine | 5.1 | |
| L-Arginine | 6.0 | |
| L-Aspartic Acid | 12.1 | |
| L- Glutamic acid | 38.2 | |
| Glycine | 3.0 | |
| L-Proline | 17.8 | |
| L-Serine | 10.0 | |
| L-Tyrosine | 9.2 | |
| Mineral Mix S10026 | 10.0 | |
| DiCalcium Phosphate | 13.0 | |
| Calcium Carbonate | 5.5 | |
| Potassium Citrate, 1 H2O | 16.5 | |
| Sodium Bicarbonate | 7.5 | |
| Vitamin Mix V10001 | 10.0 | |
| Choline Bitrartrate | 0.0 | |
| Total | 756.1 | |
|  | gm% | kcal% |
| Protein | 23.1 | 18.0 |
| Carbohydrate | 26.3 | 20.0 |
| Fat | 35.7 | 62.0 |

**Supplementary Table 2. Antibodies for analysis**

| **Antibodies** | **Source** | **Identifier** | **Dilution** |
| --- | --- | --- | --- |
| Mouse monoclonal anti-CD68 | Servicebio | Cat# GB13430 | 1:500 |
| Rabbit polyclonal anti-α-SMA | Servicebio | Cat# GB13044 | 1:1000 |
| Rabbit anti- Plin5 | Novusbio | Cat # NB110-60509 | 1:1000 |
| Mouse monoclonal anti-β-actin | Sigma-Aldrich | Cat # A5441, RRID: AB_476744 | 1:5000 |
| Mouse anti-OXPHOS | Abcam | Cat# ab110413 | 1:250 |
| Rabbit polyclonal anti-TOM20 | Cell Signaling Technology | Cat# 42406S | 1:1000 |
| Goat anti-mouse IgG secondary antibody | ZSGB-BIO, Beijing | ZB-2305 | 1:5000 |
| Goat anti-rabbit IgG secondary antibody | ZSGB-BIO, Beijing | ZB-2301 | 1:5000 |

**Supplementary Table3. Real-time PCR primers for analysis**

(H denotes human, and M denotes mice)

| **Gene** | **Direction** | **Sequence** | **Amplicon** **size (bp)** |
| --- | --- | --- | --- |
| *M- Plin5* | Forward  Reverse | GACTGAGGCTGAGCTAGCAG  GACCCCAGACGCACAAAGTA | 105bp |
| *M- β-Actin* | Forward  Reverse | AGAGGGAAATCGTGCGTGAC  CAATAGTGATGACCTGGCCGT | 138bp |
| *M- Collagen1α2* | Forward  Reverse | CCTACATGGACCAGCAGACTG  GGAGGTCTTGGTGGTTTTGTA | 182bp |
| *M- Cox7c* | Forward  Reverse | CAGAACTTCCAGCAGCGACA  CAGCAACCGCCACTTGTTTT | 133bp |
| *M-Ndufa9* | Forward  Reverse | TGAAGCAGCCGGTGTATGTT  AAGAGCAGGTACCGGTTTGG | 115bp |
| *H- PLIN5* | Forward  Reverse | TCCGGATCTCTGACCACCTT  GGCCTGAATAGGGTTCGAGG | 104bp |
| *H- β-ACTIN* | Forward  Reverse | AGCAAGCAGGAGTATGACG  AAAGGGTGTAACGCAACTAA | 89bp |

**Supplementary Table 4. The internal standard mixture for Lipidomic analyses**

| **Actual.Con.(mg/ml)** | **Compound Name** |
| --- | --- |
| 0.13 | CE(16:0)-IS |
| 0.13 | CE(16:1)-IS |
| 0.57 | CE(18:1)-IS |
| 1.43 | CE(18:2)-IS |
| 0.15 | CE(20:3)-IS |
| 0.18 | CE(20:4)-IS |
| 0.18 | CE(20:5)-IS |
| 0.22 | CE(22:6)-IS |
| 0.004 | Cer(18:0/16:0)-IS |
| 0.02 | Cer(18:1/16:0)-IS |
| 0.004 | DAG(16:0/16:0)-IS |
| 0.005 | DAG(16:0/18:0)-IS |
| 0.006 | DAG(16:0/18:1)-IS |
| 0.005 | DAG(16:0/18:2)-IS |
| 0.00135 | DAG(16:0/18:3)-IS |
| 0.0015 | DAG(16:0/20:4)-IS |
| 0.00145 | DAG(16:0/20:5)-IS |
| 0.016 | DAG(16:0/22:6)-IS |
| 0.005 | DAG(15:0/18:1)-IS |
| 0.05 | FFA(16:0)-IS |
| 0.05 | FFA(17:1)-IS |
| 0.03 | HexCer(18:1/16:0)-IS |
| 0.03 | Hex2Cer(18:1/16:0)-IS |
| 0.008522727 | LPC(16:0)-IS |
| 0.0125 | LPC(18:1)-IS |
| 0.05 | LPE(18:0)-IS |
| 0.08 | PC(15:0/18:1)-IS |
| 0.0025 | PE(15:0/18:1)-IS |
| 0.1 | SM(16:0)-IS |
| 0.1 | SM(18:1)-IS |
| 0.1 | SM(24:0)-IS |
| 0.1 | SM(24:1)-IS |
| 0.13 | TAG(50:1/FA16:0)-IS |
| 0.14 | TAG(52:1/FA18:0)-IS |
| 0.14 | TAG(52:2/FA18:1)-IS |
| 0.14 | TAG(52:3/FA18:2)-IS |
| 0.04 | TAG(52:4/FA18:3)-IS |
| 0.04 | TAG(54:4/FA20:3)-IS |
| 0.04 | TAG(54:5/FA20:4)-IS |
| 0.038 | TAG(56:7/FA22:6)-IS |
| 0.0275 | TAG(48:1/FA18:1)-IS |

**Supplementary Table 5. protein accession number for DIA proteome**

| protein name | Accessions | Description |
| --- | --- | --- |
| Ndufs5 | A0A1S6GWI4 | NADH dehydrogenase [ubiquinone] iron-sulfur protein 5 OS=Mus musculus OX=10090 GN=Ndufs5 PE=2 SV=1 |
| Ndufs4 | E9QPX3 | NADH dehydrogenase [ubiquinone] iron-sulfur protein 4, mitochondrial OS=Mus musculus OX=10090 GN=Ndufs4 PE=1 SV=1 |
| Ndufb11 | O09111 | NADH dehydrogenase [ubiquinone] 1 beta subcomplex subunit 11, mitochondrial OS=Mus musculus OX=10090 GN=Ndufb11 PE=1 SV=2 |
| Ndufb5 | Q8BU20 | NADH dehydrogenase [ubiquinone] 1 beta subcomplex subunit 5, mitochondrial OS=Mus musculus OX=10090 GN=Ndufb5 PE=2 SV=1 |
| Ndufa7 | Q9Z1P6 | NADH dehydrogenase [ubiquinone] 1 alpha subcomplex subunit 7 OS=Mus musculus OX=10090 GN=Ndufa7 PE=1 SV=3 |
| Sdhd | Q9CXV1 | Succinate dehydrogenase [ubiquinone] cytochrome b small subunit, mitochondrial OS=Mus musculus OX=10090 GN=Sdhd PE=1 SV=2 |
| Uqcrh | P99028 | Cytochrome b-c1 complex subunit 6, mitochondrial OS=Mus musculus OX=10090 GN=Uqcrh PE=1 SV=2 |
| Cox7a2l | E9PZS8 | Cytochrome c oxidase subunit 7A-related protein, mitochondrial OS=Mus musculus OX=10090 GN=Cox7a2l PE=1 SV=1 |
| ATP8 | A0A023J6C2 | ATP synthase protein 8 OS=Mus musculus musculus OX=39442 GN=ATP8 PE=3 SV=1 |
| Atp5mk | Q78IK2 | ATP synthase membrane subunit K, mitochondrial OS=Mus musculus OX=10090 GN=Atp5mk PE=1 SV=1 |
| Acad11 | A0A0R4J0I6 | Acyl-CoA dehydrogenase family member 11 OS=Mus musculus OX=10090 GN=Acad11 PE=1 SV=1 |
| Abcd1 | P48410 | ATP-binding cassette sub-family D member 1 OS=Mus musculus OX=10090 GN=Abcd1 PE=1 SV=1 |
| Abcd3 | P55096 | ATP-binding cassette sub-family D member 3 OS=Mus musculus OX=10090 GN=Abcd3 PE=1 SV=2 |
| Ehhadh | Q9DBM2 | Peroxisomal bifunctional enzyme OS=Mus musculus OX=10090 GN=Ehhadh PE=1 SV=4 |
| Crot | Q9DC50 | Peroxisomal carnitine O-octanoyltransferase OS=Mus musculus OX=10090 GN=Crot PE=1 SV=1 |
| Acox1 | Q9R0H0 | Peroxisomal acyl-coenzyme A oxidase 1 OS=Mus musculus OX=10090 GN=Acox1 PE=1 SV=5 |
|  |  |  |

**Supplementary Table 6 Contents of hepatic TG in CDAA diet-induced MASH mice**

| Name of TGs | CD1 | CD2 | CD3 | CD4 | CD5 | CDAA1 | CDAA2 | CDAA3 | CDAA4 | CDAA5 | CDAA+DDC1 | CDAA+DDC2 | CDAA+DDC3 | CDAA+DDC4 | CDAA+DDC5 |
| --- | --- | --- | --- | --- | --- | --- | --- | --- | --- | --- | --- | --- | --- | --- | --- |
| TG(12:0/12:0/16:0) | 0.325445094 | 0.134935937 | 0.378510051 | 0.301867339 | 0.280985165 | 0.792393299 | 2.752522434 | 1.039245639 | 1.345630092 | 1.1537366 | 2.084550826 | 1.538565577 | 2.04928158 | 1.507195194 | 1.721062064 |
| TG(12:0/14:0/16:1) | 0.333147757 | 0.112723649 | 0.44593649 | 0.406765976 | 0.336836011 | 1.636469693 | 5.23437479 | 2.412439583 | 3.859032118 | 2.532626839 | 5.59450766 | 4.872927586 | 6.476866988 | 4.512480572 | 4.880641887 |
| TG(12:0/14:0/16:0) | 0.404694563 | 0.15933517 | 0.505821086 | 0.457227665 | 0.362922289 | 0.747703859 | 1.808268488 | 0.866136014 | 1.284992304 | 1.192939442 | 2.103055203 | 1.740948324 | 3.317613259 | 1.570415072 | 1.723746623 |
| TG(12:0/16:1/16:1) | 1.08555891 | 0.283843464 | 1.471776796 | 1.130866914 | 0.039345898 | 5.634306914 | 20.55829954 | 7.14430188 | 15.51648831 | 9.095604613 | 21.51676586 | 19.77892892 | 28.40495515 | 16.5983776 | 18.90911763 |
| TG(14:0/16:1/14:0) | 1.726167842 | 0.432275751 | 2.250778371 | 1.847973375 | 1.515743289 | 4.783989737 | 13.69856556 | 6.773882847 | 15.65854114 | 7.790040668 | 15.89265079 | 13.3952425 | 17.87663087 | 10.97946875 | 15.32758136 |
| TG(14:1/16:1/16:1) | 3.910043418 | 0.619106395 | 5.513985145 | 4.129986312 | 3.825060971 | 16.25300458 | 64.02586636 | 23.57204807 | 49.07363948 | 29.8111831 | 73.27121825 | 62.69840019 | 89.04683614 | 50.14262837 | 35.63159455 |
| TG(12:0/18:1/16:1) | 10.19957168 | 1.904965371 | 13.76255943 | 10.76170143 | 9.496154161 | 31.31245164 | 96.25184115 | 41.7070114 | 84.33291114 | 51.40981186 | 109.2349221 | 97.12605592 | 122.2919127 | 77.39288274 | 94.51590049 |
| TG(14:0/16:0/16:1) | 9.211049822 | 2.530591542 | 10.58724673 | 9.094806145 | 7.972433283 | 18.16641143 | 45.15918753 | 23.91436276 | 41.91638015 | 28.24138339 | 58.34450463 | 52.83266489 | 62.38558736 | 41.73543264 | 46.63132804 |
| TG(14:0/16:0/16:0) | 3.682362568 | 1.374815287 | 4.00897968 | 2.954589042 | 3.546783458 | 5.239998131 | 11.5802615 | 6.743748873 | 10.90988671 | 7.827439326 | 17.72745031 | 16.22254403 | 17.453034 | 12.40826651 | 13.36008244 |
| TG(16:1/16:1/16:1) | 32.32875938 | 6.456921653 | 40.72917209 | 31.90958763 | 30.46059765 | 90.99771514 | 259.5133344 | 113.4695881 | 221.5137588 | 138.5751068 | 294.7486465 | 279.5079735 | 334.1921712 | 221.4349004 | 252.5312533 |
| TG(14:0/16:1/18:1) | 59.66481896 | 16.83004679 | 66.61514437 | 58.73726173 | 49.34582274 | 119.5166553 | 305.2716484 | 137.0371521 | 241.4123233 | 148.696985 | 324.0727261 | 310.4308654 | 333.562528 | 279.7093836 | 271.5471832 |
| TG(14:0/16:0/18:1) | 45.06108756 | 16.15675844 | 56.71672923 | 53.49884275 | 40.27830865 | 82.3214046 | 187.4041058 | 97.34048756 | 176.9235991 | 114.9400866 | 251.0754205 | 242.506796 | 256.8132314 | 174.4690932 | 199.1049053 |
| TG(16:0/16:0/16:0) | 16.40197379 | 6.225235913 | 21.03921904 | 14.55969897 | 13.54844188 | 29.29634961 | 71.35840291 | 35.33789749 | 62.34118641 | 38.67075092 | 127.2466156 | 80.60844047 | 88.40189486 | 57.07216633 | 59.33545353 |
| TG(16:1/16:1/17:1) | 4.45113784 | 1.427881534 | 6.005675921 | 4.98829771 | 5.383252523 | 14.67498317 | 26.95876445 | 12.32588456 | 22.99822572 | 13.60186035 | 41.25888613 | 24.35647356 | 34.26644036 | 25.99225537 | 22.41200322 |
| TG(16:0/16:0/17:2) | 7.18627028 | 3.449228514 | 8.446193253 | 6.827558395 | 6.331148031 | 16.30496111 | 33.13019791 | 16.38323324 | 34.67914707 | 17.25574616 | 36.03400279 | 33.69611913 | 34.94053832 | 24.42737828 | 30.36401821 |
| TG(16:0/16:0/17:1) | 7.790200708 | 2.663056694 | 9.261104032 | 7.903446982 | 6.113222408 | 13.86791895 | 33.44021045 | 18.1064558 | 34.18817538 | 20.77856618 | 40.18600066 | 39.79536697 | 40.08469818 | 27.24653577 | 34.11445706 |
| TG(16:0/16:0/17:0) | 2.189848054 | 0.750810045 | 2.516855395 | 1.899734226 | 1.197066386 | 3.842544194 | 11.1305161 | 4.849214836 | 9.171263609 | 6.251202546 | 13.26543919 | 13.2519057 | 14.70420898 | 8.473998224 | 10.49681412 |
| TG(16:0/16:1/18:3) | 122.613001 | 23.96172914 | 134.125595 | 102.2900808 | 5.746817921 | 255.938055 | 790.4974962 | 336.6913534 | 700.6054903 | 423.1186614 | 861.341646 | 969.2818293 | 1043.569608 | 673.4616813 | 758.8049801 |
| TG(16:1/16:1/18:1) | 0.052262055 | 0.017220044 | 0.036617024 | 0.049697648 | 0.031715676 | 0.116326702 | 1.086316539 | 0.167366315 | 0.2932756 | 0.165142249 | 0.419798988 | 0.308694856 | 0.490470489 | 0.255877203 | 0.308377208 |
| TG(16:0/16:1/18:2) | 324.730646 | 94.91767222 | 352.3144882 | 300.2494431 | 19.73686889 | 587.7747404 | 1493.059795 | 748.7135357 | 1462.616468 | 850.2701871 | 1677.275091 | 1772.068954 | 1782.598176 | 1216.958708 | 1497.638562 |
| TG(16:0/16:0/18:2) | 8.313166598 | 2.375550073 | 9.833202814 | 7.689577082 | 6.638658191 | 14.78797072 | 43.95062938 | 22.05183053 | 42.78169056 | 24.2408169 | 55.84233755 | 31.16864757 | 55.27752585 | 33.44401366 | 40.46840335 |
| TG(16:0/16:0/18:1) | 344.1024152 | 142.9094507 | 411.5584803 | 318.7965798 | 57.32746381 | 589.0098015 | 1440.478968 | 779.8625039 | 1412.247188 | 855.1397252 | 1932.703129 | 1577.222543 | 1683.658729 | 1323.361575 | 1252.345572 |
| TG(16:0/18:0/16:0) | 34.01032501 | 11.41538056 | 45.01553622 | 32.01403796 | 29.82607576 | 65.5781679 | 183.4472067 | 83.28693471 | 153.6818978 | 96.63109541 | 230.7743139 | 213.9924481 | 249.3033755 | 154.0240134 | 174.0127919 |
| TG(16:1/17:2/18:3) | 3.846746151 | 0.192899635 | 1.415967927 | 1.355628899 | 1.379601155 | 6.749614294 | 17.99981041 | 14.959223 | 30.06940709 | 17.544181 | 18.79876069 | 32.86424301 | 19.16110125 | 16.80993272 | 33.66605153 |
| TG(17:2/17:2/17:2) | 0.451454419 | 0.047250428 | 0.925275972 | 0.726174536 | 0.367966443 | 8.209807319 | 22.51121049 | 11.76490791 | 13.7025211 | 9.117528395 | 16.6928252 | 13.90225877 | 31.4148643 | 13.42099249 | 14.71882415 |
| TG(17:1/17:2/17:2) | 4.856765932 | 0.891915101 | 6.304351944 | 8.20912811 | 5.029640386 | 9.101784438 | 45.03847859 | 19.59565994 | 35.83849149 | 20.19003912 | 70.16594481 | 47.43876695 | 52.12733215 | 40.8693492 | 39.18080234 |
| TG(16:0/17:2/18:3) | 1.296707741 | 0.72669125 | 1.229014625 | 1.181533053 | 0.975033157 | 1.499917245 | 2.193144241 | 1.562710857 | 2.650551732 | 1.936833388 | 2.60315873 | 2.794421074 | 2.303612631 | 1.779505447 | 1.933486763 |
| TG(16:0/17:1/18:3) | 14.38824054 | 3.814097949 | 16.12744196 | 13.22242384 | 2.945162497 | 30.79851485 | 84.12541074 | 39.0936084 | 71.12495178 | 41.15591795 | 90.34841931 | 86.9639826 | 102.480498 | 67.88994427 | 73.64579975 |
| TG(17:0/17:2/17:2) | 0.298193495 | 0.148080391 | 0.304559527 | 0.256804627 | 0.271636866 | 0.810597424 | 1.674515093 | 0.96168744 | 1.020311191 | 0.946935723 | 0.827471974 | 0.848922361 | 1.356312062 | 1.190545315 | 0.816007918 |
| TG(17:1/17:1/17:1) | 36.99967839 | 10.97886501 | 40.78594896 | 34.73565037 | 28.84629076 | 67.14934876 | 173.8157184 | 97.44928689 | 176.9201202 | 97.5181027 | 198.1206963 | 213.608593 | 223.466023 | 142.0375847 | 165.7695612 |
| TG(17:0/17:1/17:1) | 2.663212085 | 0.745027708 | 2.705483335 | 2.777483807 | 2.793656942 | 13.2049294 | 27.59167077 | 17.37575104 | 20.72238593 | 14.88770441 | 19.15204335 | 18.31726312 | 25.17595952 | 20.22570885 | 18.76707863 |
| TG(16:0/17:0/18:1) | 28.16003863 | 6.949340314 | 33.77791325 | 35.15198758 | 21.88993378 | 57.29100122 | 176.1092783 | 78.99792498 | 166.0213454 | 86.76380633 | 201.2228522 | 205.3455467 | 223.2984127 | 126.6532957 | 161.8571261 |
| TG(17:0/17:0/17:1) | 0.556362532 | 0.156824846 | 0.544268814 | 0.602107858 | 0.542391864 | 2.514108947 | 5.64794405 | 3.415935595 | 4.134439967 | 3.025732114 | 4.103415269 | 4.020374232 | 5.460339612 | 4.043644389 | 4.207765275 |
| TG(16:1/18:3/18:3) | 23.06925397 | 2.917603638 | 27.55578663 | 21.97264889 | 20.37472132 | 78.31578172 | 362.8499125 | 112.1583789 | 274.1114678 | 149.2711967 | 367.2728814 | 387.6934199 | 463.1697277 | 277.6941124 | 300.7314593 |
| TG(17:0/17:0/17:0) | 0.32067444 | 0.09859633 | 0.391319644 | 0.403388893 | 0.378367517 | 1.98477878 | 5.578487111 | 3.071254372 | 3.913403182 | 2.486904763 | 4.027110409 | 3.763642876 | 5.450580057 | 3.641151245 | 3.82745323 |
| TG(16:1/18:2/18:3) | 129.6420618 | 20.07575858 | 153.2019273 | 116.6872023 | 2.741995758 | 343.5104613 | 1303.899559 | 550.0172184 | 1250.729886 | 620.2330297 | 1354.887929 | 1678.717958 | 1963.26549 | 1154.679614 | 1335.54274 |
| TG(16:0/18:2/18:3) | 659.7799679 | 159.1229698 | 786.5054821 | 617.4781076 | 12.75451428 | 1365.893019 | 4192.781023 | 1858.748838 | 3645.9721 | 2191.88998 | 4339.681493 | 4295.823315 | 4996.260906 | 3112.161648 | 3731.323688 |
| TG(16:0/18:2/18:2) | 1754.212985 | 565.1251554 | 1959.12532 | 1608.968512 | 1427.811197 | 3213.265264 | 8120.814133 | 4229.359277 | 7549.919445 | 4534.421398 | 8459.543687 | 8683.391724 | 9363.451555 | 6029.235635 | 7101.473749 |
| TG(16:0/18:1/18:2) | 2262.644852 | 838.6913493 | 2545.838124 | 2154.34357 | 1928.171981 | 4637.013454 | 9079.116538 | 5150.105022 | 8328.593849 | 5411.387225 | 9224.324449 | 9440.932705 | 10266.42532 | 6657.688899 | 10026.74818 |
| TG(18:1/16:0/18:1) | 45.33155141 | 10.92761852 | 54.62661492 | 39.53946498 | 31.51685104 | 96.41217274 | 393.7226499 | 175.4685386 | 383.1087663 | 177.6588491 | 362.477861 | 409.0790587 | 471.1569152 | 265.3508474 | 336.6838044 |
| TG(16:0/18:0/18:1) | 541.3654335 | 183.6438457 | 710.255512 | 556.9549179 | 484.5439281 | 1085.026395 | 3103.565486 | 1492.133077 | 2711.27932 | 1686.474857 | 3374.006649 | 3513.880036 | 3961.126283 | 2407.467071 | 2926.640803 |
| TG(17:1/18:3/18:3) | 3.770739398 | 0.769418408 | 5.223807663 | 4.489066461 | 4.206135845 | 20.61448747 | 59.92937137 | 14.4651458 | 30.26806749 | 20.56771808 | 60.32721294 | 33.39186523 | 67.11235506 | 16.74923709 | 27.7557104 |
| TG(16:0/18:0/18:0) | 27.57544277 | 7.243936948 | 38.35658476 | 27.82392773 | 26.08597071 | 59.44468507 | 178.4406304 | 81.75782558 | 161.6667503 | 96.0059457 | 285.4488324 | 259.6359662 | 317.3735698 | 175.8051676 | 211.0811986 |
| TG(17:0/18:3/18:3) | 7.024538635 | 4.170605203 | 7.798737855 | 6.776194852 | 1.052703731 | 8.685158333 | 13.70654679 | 11.0351194 | 15.27930277 | 10.19204273 | 13.83295842 | 15.9039612 | 14.52051588 | 10.77755587 | 11.52970715 |
| TG(17:2/18:2/18:2) | 1.127305041 | 0.131402849 | 2.029886629 | 1.46024079 | 0.989574132 | 10.4409728 | 31.0212984 | 23.87714905 | 27.19145363 | 18.33977597 | 35.62645299 | 27.74286772 | 44.00739537 | 16.66404411 | 28.91811133 |
| TG(17:1/18:2/18:3) | 3.842526125 | 1.307586192 | 3.921753943 | 2.964398212 | 3.166484124 | 5.530102833 | 13.62766359 | 4.560452043 | 12.21164048 | 4.026980282 | 15.38411813 | 15.12697382 | 16.34249689 | 10.2506622 | 10.80118391 |
| TG(16:1/17:2/20:3) | 0.575908488 | 0.048864051 | 0.988821929 | 0.817836933 | 0.484397698 | 7.591613948 | 23.72151603 | 21.39502467 | 16.58088592 | 11.83890021 | 26.01150019 | 18.53210341 | 30.23918391 | 11.54182366 | 18.57338758 |
| TG(17:1/18:2/18:2) | 15.87528396 | 4.148413425 | 17.8056727 | 13.785163 | 12.78001728 | 32.19585378 | 87.79932548 | 42.76311848 | 77.21023855 | 47.28247689 | 92.53435828 | 96.1239745 | 104.6904435 | 68.6332757 | 78.69068285 |
| TG(16:1/17:1/20:3) | 4.710825855 | 2.274554368 | 5.248084784 | 4.28312747 | 3.734072205 | 5.374484127 | 9.250454257 | 6.865208962 | 10.65236858 | 7.02172653 | 10.06794138 | 11.55910855 | 10.18817015 | 7.795202794 | 10.59065425 |
| TG(17:0/18:2/18:2) | 34.62622584 | 9.94348321 | 36.59530396 | 30.62961985 | 3.566707133 | 62.29757597 | 161.8216413 | 85.5134731 | 150.2303943 | 92.33811176 | 181.663628 | 189.452873 | 203.4121051 | 129.6330187 | 157.1920851 |
| TG(17:0/18:1/18:2) | 1.256149656 | 0.346639094 | 1.316118755 | 1.439009386 | 1.292376529 | 5.329775425 | 14.57089508 | 8.3702779 | 11.75921517 | 7.181849742 | 9.569480783 | 10.05939231 | 12.77356157 | 8.51449023 | 9.801688013 |
| TG(17:1/17:1/19:0) | 88.81184757 | 20.21549696 | 102.4524054 | 84.37263649 | 70.01482403 | 230.7254437 | 615.4737666 | 290.5799873 | 602.8772032 | 313.6983919 | 638.8768229 | 676.3745169 | 780.4050087 | 429.26216 | 561.5571114 |
| TG(17:0/18:1/18:1) | 1.5478009 | 0.404018286 | 1.906679334 | 2.169162549 | 1.661118151 | 9.481581206 | 34.59748465 | 17.92321247 | 2.368176208 | 13.18024352 | 20.57493967 | 20.31398555 | 28.59495376 | 16.14988738 | 21.51483559 |
| TG(16:1/18:2/20:5) | 53.87281668 | 5.882133705 | 53.01578593 | 39.41809257 | 37.69252564 | 126.968079 | 584.1589535 | 176.4986479 | 416.5693461 | 243.5969666 | 891.3966014 | 1006.50101 | 742.0088395 | 405.7596579 | 462.3206705 |
| TG(16:0/18:1/19:0) | 16.8275486 | 3.503319023 | 20.75339485 | 16.55223831 | 12.26051424 | 42.05588228 | 154.8077228 | 66.19118633 | 145.3138597 | 79.6494926 | 193.8428126 | 194.363074 | 362.7429576 | 114.3760567 | 158.2418025 |
| TG(18:1/18:3/18:3) | 260.7354091 | 50.5364192 | 283.9168049 | 225.6001996 | 4.905152525 | 556.0544165 | 2055.288397 | 795.9532523 | 1757.534289 | 898.8908023 | 1810.669471 | 2019.223138 | 2180.042155 | 1181.879695 | 1626.889048 |
| TG(17:0/18:0/18:0) | 0.654820909 | 0.172265158 | 0.94081453 | 0.693635723 | 2.507743928 | 1.79851542 | 7.766257543 | 2.620061006 | 5.278970796 | 3.461711719 | 11.57630671 | 8.636840795 | 11.51206229 | 6.282558759 | 7.228291046 |
| TG(18:2/18:2/18:2) | 0.257828313 | 0.057138961 | 0.30919793 | 0.21915781 | 0.144804605 | 0.243081739 | 2.630982756 | 0.846303042 | 1.681636233 | 1.210612097 | 2.786214066 | 2.485391775 | 3.292596538 | 1.725716792 | 2.189504908 |
| TG(18:1/18:2/18:2) | 1.607256269 | 0.408625675 | 1.929203637 | 1.422515725 | 1.340217877 | 3.371267577 | 9.71332376 | 4.698194577 | 8.623181502 | 26.11058393 | 9.000005885 | 10.13761876 | 11.97224466 | 6.804863042 | 8.350531338 |
| TG(16:0/18:1/20:4) | 984.8389471 | 259.0417152 | 1029.341197 | 946.0874372 | 775.8599677 | 1992.02336 | 5370.81083 | 2729.742789 | 4798.846323 | 3258.21518 | 5642.804577 | 5552.086684 | 5892.894503 | 3740.346886 | 4635.87148 |
| TG(18:1/18:1/18:3) | 1.591584332 | 0.447576472 | 1.683594833 | 1.492770643 | 1.189934532 | 3.155010451 | 9.933880308 | 4.553141544 | 8.440324706 | 4.703891706 | 10.17194351 | 10.90491784 | 12.29132766 | 6.145558657 | 8.444609811 |
| TG(18:0/18:2/18:2) | 1.536897873 | 0.318642881 | 1.995124716 | 1.811618386 | 1.467999328 | 4.592045407 | 16.9592367 | 7.752502676 | 13.14061433 | 7.583449118 | 16.22627472 | 17.63450616 | 22.00677565 | 11.17675365 | 14.09756427 |
| TG(16:0/18:1/20:3) | 1622.018145 | 530.363223 | 1803.620225 | 1490.155675 | 262.0758891 | 2931.59138 | 6674.709905 | 3748.710149 | 5811.822017 | 4250.354433 | 7167.69709 | 7228.721791 | 7975.112867 | 5448.198439 | 6095.062258 |
| TG(18:1/18:1/18:1) | 1337.006182 | 445.2033489 | 1900.905308 | 1292.653116 | 1202.949549 | 2655.414631 | 6355.89231 | 3436.900975 | 5350.357326 | 3610.843276 | 6491.56288 | 6498.808193 | 7213.453468 | 4999.517548 | 5371.549543 |
| TG(16:0/18:2/20:1) | 0.634803036 | 0.119573065 | 0.623348413 | 0.497173419 | 0.451779942 | 1.665466028 | 5.82041006 | 2.344879743 | 4.890781457 | 3.098507268 | 6.866512636 | 7.186150149 | 10.09424221 | 4.1414915 | 5.996826618 |
| TG(17:2/18:3/20:4) | 6.498056319 | 3.351079937 | 6.471354582 | 5.412067584 | 5.6009142 | 8.404418954 | 12.12360605 | 9.401396157 | 20.77622938 | 10.95982106 | 16.67937649 | 14.82981712 | 14.42182118 | 11.99811486 | 12.81318041 |
| TG(18:0/18:0/18:2) | 763.8020034 | 244.8379048 | 968.5356032 | 777.885479 | 731.3278893 | 1740.574241 | 4824.127051 | 2348.23385 | 3977.430373 | 3267.522963 | 5046.617469 | 4978.480115 | 5980.904585 | 3695.544316 | 4289.095357 |
| TG(17:1/18:2/20:5) | 7.35425443 | 3.801522421 | 7.643060449 | 6.421083832 | 0.178222975 | 8.064993911 | 13.10082741 | 10.4536654 | 18.52352213 | 10.47836467 | 20.23631035 | 19.0164982 | 14.14029105 | 11.65892573 | 12.74595257 |
| TG(16:0/18:1/20:0) | 1.25857165 | 0.450979127 | 2.546762884 | 1.138776257 | 2.083996394 | 2.091802875 | 4.626009726 | 2.444187315 | 3.784694412 | 3.025368416 | 5.716666589 | 4.988861538 | 6.286323128 | 3.809348672 | 4.162703573 |
| TG(16:1/18:0/20:0) | 0.05965644 | 0.018804895 | 0.061693899 | 0.060303856 | 0.045587728 | 0.32465901 | 1.154475274 | 0.540048148 | 0.538356941 | 0.417020921 | 0.593936523 | 0.80819921 | 0.876633849 | 0.349817479 | 0.619449583 |
| TG(17:1/18:1/20:5) | 13.42981033 | 3.109645422 | 14.09195494 | 11.92736168 | 10.1030084 | 38.40800301 | 83.9314197 | 41.51158547 | 98.15045982 | 41.1097259 | 78.52936233 | 88.81626211 | 126.2321583 | 57.960921 | 68.67385529 |
| TG(17:1/18:2/20:4) | 7.833860277 | 4.251894622 | 8.810252528 | 7.258773377 | 7.183036648 | 10.53331266 | 17.30243921 | 11.21658669 | 14.90112529 | 11.625702 | 18.98450675 | 17.8903638 | 18.01934888 | 14.14074488 | 15.32337638 |
| TG(18:0/18:0/18:0) | 5.316756965 | 1.692883505 | 10.25972556 | 6.444123921 | 0.234233442 | 17.16758919 | 76.2960051 | 21.5621202 | 43.89517482 | 29.19435724 | 119.4255815 | 80.67354352 | 118.9427016 | 70.75494686 | 72.39480933 |
| TG(17:0/18:1/20:5) | 26.1476008 | 5.776449292 | 28.21319157 | 23.69881881 | 15.8936801 | 36.41418969 | 95.11979841 | 52.8708587 | 82.52128081 | 85.263858 | 89.90087042 | 96.13405984 | 100.7440007 | 68.58347821 | 120.8185191 |
| TG(17:1/18:2/20:3) | 8.284647177 | 4.193227807 | 9.737042483 | 8.01419541 | 7.156482609 | 10.57494614 | 19.70846976 | 19.0147232 | 18.62142769 | 13.97571595 | 33.23170141 | 23.25111348 | 21.97476687 | 15.89374668 | 20.6303569 |
| TG(16:0/17:2/22:4) | 0.964632472 | 0.531153629 | 0.75155204 | 0.56513473 | 1.071062349 | 2.956278568 | 4.616549147 | 3.041415695 | 4.283920654 | 3.648123927 | 4.394951316 | 3.394042398 | 4.948331203 | 3.953946458 | 3.83170703 |
| TG(17:1/18:1/20:3) | 11.75249588 | 3.651604138 | 12.9274 | 12.13749698 | 9.121979189 | 43.7442422 | 55.66018896 | 31.31690226 | 55.04201333 | 69.73007579 | 55.8241986 | 59.5110101 | 59.61890221 | 43.41566444 | 50.62094878 |
| TG(18:2/18:2/19:0) | 21.08745887 | 5.987331667 | 23.47623473 | 19.41411045 | 17.12660213 | 41.73682856 | 115.1025765 | 58.00196714 | 94.08974755 | 63.35912758 | 123.7527769 | 125.1964099 | 139.3258279 | 87.13965308 | 101.9189581 |
| TG(16:0/17:0/22:4) | 4.31386728 | 1.259064551 | 5.166968301 | 4.15759173 | 3.278512797 | 8.648472085 | 25.30784366 | 11.31828765 | 20.14220068 | 13.66852686 | 25.62258818 | 25.12259458 | 29.65271926 | 16.53891064 | 20.74784004 |
| TG(16:1/20:4/20:5) | 12.33559272 | 1.306145273 | 12.39147007 | 8.363633202 | 8.46405538 | 27.17924628 | 159.9995434 | 35.47566419 | 117.1370177 | 61.05510604 | 165.2992635 | 198.3441549 | 190.970827 | 118.2185498 | 133.1605223 |
| TG(17:0/18:2/20:1) | 26.97386395 | 6.405728994 | 33.57148888 | 26.97463406 | 4.250785504 | 65.34523782 | 205.3518392 | 99.70909684 | 178.1756694 | 110.2302675 | 221.6780285 | 223.0514538 | 262.3489838 | 154.2600649 | 185.0522549 |
| TG(16:0/17:1/22:2) | 17.35135552 | 6.091435077 | 17.20088062 | 16.47273324 | 14.16400949 | 23.09740297 | 53.45320851 | 27.4504902 | 47.81092276 | 34.21024366 | 56.1618421 | 61.32552304 | 68.40989127 | 42.65848868 | 49.60555061 |
| TG(16:1/20:4/20:4) | 224.2224496 | 28.95503153 | 240.0933066 | 91.32735021 | 1.825000909 | 320.0027766 | 1505.543873 | 478.2343007 | 1064.2805 | 606.9898478 | 1552.178903 | 1740.732585 | 1904.525371 | 1103.699431 | 1095.911812 |
| TG(18:1/18:1/19:0) | 14.015244 | 3.193992482 | 18.82293508 | 15.11592074 | 12.77699776 | 42.36999286 | 161.0644697 | 68.34007481 | 132.5927371 | 81.47203407 | 185.9699413 | 169.259614 | 208.3604236 | 113.5040889 | 140.2591405 |
| TG(17:2/19:0/19:0) | 1.688836548 | 0.391388429 | 1.991586734 | 2.770251882 | 1.69957876 | 13.00476279 | 53.2333987 | 25.45971375 | 32.90945754 | 18.24840998 | 25.77035198 | 24.00748106 | 38.7291468 | 22.7217511 | 28.59858775 |
| TG(16:0/18:2/22:6) | 1309.955084 | 216.076259 | 1027.787034 | 802.7599981 | 697.8001984 | 2611.531266 | 5814.316828 | 2427.504694 | 4672.59897 | 2789.696704 | 5247.942319 | 5757.494987 | 4696.53842 | 3370.674883 | 4641.770492 |
| TG(18:0/18:1/19:0) | 2.251284231 | 0.659302558 | 3.806692401 | 2.599390631 | 0.167615369 | 7.860712063 | 37.92103374 | 11.40269552 | 24.44771376 | 15.56783354 | 50.5551631 | 35.37294581 | 49.56066174 | 26.58744013 | 30.71092699 |
| TG(18:1/18:2/20:4) | 1.494903932 | 0.491402216 | 1.952557973 | 1.433299672 | 1.293743116 | 2.94423458 | 7.891127976 | 3.61054109 | 6.201438461 | 4.194721982 | 7.345870197 | 8.8869876 | 9.401525036 | 5.296111168 | 5.974106129 |
| TG(17:0/19:0/19:0) | 3.160016424 | 0.947468606 | 3.832612539 | 109.7389778 | 90.91701251 | 6.047657417 | 12.97174934 | 8.343487099 | 10.18051567 | 458.8717298 | 13.17395707 | 12.29092874 | 12.81160653 | 13.82051796 | 458.4126268 |
| TG(18:1/18:1/20:4) | 4.975127897 | 1.176089194 | 5.736382942 | 4.723884533 | 4.013709524 | 13.32662267 | 33.31141599 | 16.1880049 | 25.05264938 | 18.273751 | 26.83793314 | 30.66958663 | 33.63414901 | 19.38542843 | 25.74428612 |
| TG(16:0/18:1/22:4) | 0.321180251 | 0.07459396 | 0.335976278 | 0.28673812 | 0.300258881 | 1.030078324 | 4.676511134 | 1.804489544 | 3.652765168 | 1.936475092 | 5.782451403 | 4.546482312 | 6.636706155 | 2.524956778 | 3.874064918 |
| TG(18:1/18:1/20:3) | 0.143329579 | 0.043006265 | 0.155080342 | 0.158936323 | 0.117920218 | 0.559492597 | 1.457309378 | 0.728858446 | 1.013986398 | 0.7095573 | 1.718405509 | 1.55198457 | 2.036100848 | 0.885130307 | 1.422341951 |
| TG(18:1/18:1/20:2) | 369.280939 | 213.5575333 | 473.6164189 | 723.200366 | 193.0464026 | 1661.852679 | 2711.602192 | 1229.993239 | 2121.581175 | 2529.497221 | 2643.747045 | 2347.577867 | 2905.043361 | 3545.822153 | 2191.277219 |
| TG(17:2/20:4/20:4) | 3.203169799 | 0.282328731 | 2.247988797 | 1.912656906 | 0.899419188 | 5.626045507 | 40.01625535 | 8.18267079 | 15.91765911 | 10.47862367 | 19.34737785 | 20.76443768 | 22.84672123 | 24.4691636 | 16.51534917 |
| TG(17:2/18:2/22:6) | 12.12903483 | 6.565863866 | 10.96075763 | 9.877641305 | 0.331230235 | 13.36916028 | 25.35302635 | 15.06187818 | 19.3531915 | 15.78709527 | 30.79989503 | 34.22451237 | 20.91304409 | 16.43048906 | 18.65794193 |
| TG(18:1/18:1/20:1) | 215.8658433 | 71.38312487 | 404.0056223 | 239.9715529 | 68.76685576 | 623.9020445 | 1953.533864 | 839.5792927 | 1408.063022 | 923.2635883 | 1996.311191 | 1612.927977 | 2137.454164 | 1374.68139 | 1544.469146 |
| TG(17:1/18:2/22:6) | 7.64342767 | 1.124489812 | 6.027435325 | 5.238923356 | 4.513304624 | 13.13435548 | 43.89521133 | 19.71781951 | 35.8092471 | 23.13722744 | 44.39808691 | 48.17381786 | 47.2220593 | 31.59673486 | 39.2576823 |
| TG(17:2/18:2/22:5) | 10.52149633 | 6.296157271 | 10.76410397 | 10.43117291 | 9.565552464 | 14.78776078 | 24.93669241 | 15.44438728 | 22.68969453 | 18.10278081 | 25.56396329 | 34.27649207 | 24.90510555 | 25.13629272 | 22.90847178 |
| TG(18:0/18:1/20:1) | 0.0706351 | 0.030259327 | 0.756696946 | 0.101417887 | 0.081626074 | 0.359989368 | 1.031131394 | 0.342289047 | 0.715637225 | 0.465088011 | 1.076743762 | 0.743152508 | 1.358365504 | 9.202470392 | 0.662602392 |
| TG(18:1/18:1/20:0) | 0.056717219 | 0.011084796 | 0.055559833 | 0.050183703 | 0.043211904 | 0.321284894 | 1.226969331 | 0.590270476 | 0.576379285 | 0.375331584 | 0.536474636 | 0.633234228 | 1.035716647 | 0.363886208 | 0.663959951 |
| TG(17:0/20:4/20:4) | 5.048980286 | 2.581668249 | 6.119845507 | 5.301983323 | 4.869422255 | 7.601745721 | 20.95131063 | 9.449840994 | 12.51236227 | 14.39332328 | 14.3161761 | 12.59326853 | 15.6670509 | 11.81015681 | 18.96120389 |
| TG(16:0/18:1/22:0) | 5.273769061 | 2.087928641 | 6.252564491 | 3.748356752 | 4.025095889 | 7.83832386 | 21.24987518 | 8.045220019 | 16.73451335 | 11.96635864 | 20.66822302 | 21.91906143 | 24.31717186 | 15.26827079 | 18.24726614 |
| TG(18:0/18:0/20:1) | 0.031075572 | 0.011338576 | 0.045745548 | 0.028659203 | 0.027443069 | 0.151821046 | 0.589389638 | 0.247967803 | 0.253867969 | 0.233653617 | 0.423502003 | 0.314281943 | 0.560097409 | 0.355296413 | 0.317022529 |
| TG(17:1/20:2/20:4) | 10.61336894 | 3.07332023 | 16.3522118 | 14.19118422 | 12.44974773 | 30.35580844 | 73.45892962 | 40.46231549 | 63.76627817 | 45.35010237 | 73.46384511 | 77.37909058 | 78.27963271 | 54.39468292 | 66.07428038 |
| TG(16:1/19:0/22:6) | 2.533341063 | 0.327733424 | 4.250577583 | 3.146881507 | 2.038649533 | 26.23000476 | 80.79058687 | 54.84416612 | 55.74257856 | 39.40740987 | 88.03380751 | 65.44957639 | 111.5282841 | 40.48798789 | 71.94096779 |
| TG(17:0/18:1/22:5) | 17.43843736 | 3.968363451 | 19.89529332 | 18.10359442 | 10.69362657 | 26.58089609 | 67.27545243 | 34.81079248 | 57.54226233 | 42.70277716 | 64.5413468 | 65.18382603 | 69.46972557 | 47.8723007 | 56.99507219 |
| TG(18:0/19:0/20:5) | 33.13229193 | 8.59116784 | 38.15906213 | 31.24894036 | 28.18099644 | 58.22593534 | 129.9639041 | 73.85150119 | 130.6953337 | 83.12276388 | 140.958045 | 141.1129478 | 154.9976225 | 106.1972664 | 125.2939244 |
| TG(18:3/18:3/22:5) | 41.52418305 | 5.993312613 | 45.39763829 | 24.66595742 | 0.920727338 | 107.8812351 | 687.3917718 | 192.9519505 | 361.4576064 | 303.7683382 | 652.0619415 | 744.978687 | 792.2224496 | 439.5959771 | 424.711869 |
| TG(17:0/20:2/20:2) | 5.772297729 | 1.553973991 | 7.67331596 | 5.758014463 | 5.151296984 | 26.33521903 | 45.80346 | 20.57657741 | 36.7110883 | 24.38006833 | 46.57965611 | 72.47974738 | 51.41244175 | 33.46809476 | 35.01873932 |
| TG(18:2/19:0/20:2) | 59.61900313 | 25.9693015 | 65.95214102 | 56.15725549 | 48.77144841 | 88.03908216 | 202.4614262 | 103.8000431 | 184.9415494 | 121.3272579 | 210.4730123 | 213.4626716 | 357.8625141 | 152.2271342 | 270.535443 |
| TG(18:2/18:2/22:6) | 294.9548983 | 42.30361392 | 329.0602445 | 216.5300966 | 217.6812715 | 702.0769237 | 2936.28786 | 1030.060913 | 1896.879588 | 1238.430158 | 2767.860958 | 3098.729231 | 3321.110926 | 1889.599086 | 1977.654191 |
| TG(17:0/20:1/20:2) | 3.680944275 | 0.999984017 | 5.767214567 | 4.10870686 | 0.152598396 | 13.14236843 | 49.25361175 | 20.12687564 | 37.9324933 | 23.15877387 | 55.24194322 | 43.98601598 | 59.49289793 | 34.38247728 | 39.01418688 |
| TG(18:2/18:2/22:5) | 726.8057256 | 158.5244766 | 802.2561654 | 608.387952 | 577.1413433 | 1520.845814 | 4711.570944 | 2059.788186 | 3446.18238 | 2433.490094 | 4444.471331 | 4468.169277 | 4767.42393 | 3068.071352 | 3632.552465 |
| TG(18:1/18:1/21:0) | 1.880508284 | 0.582165756 | 3.348030666 | 2.29761378 | 3.92138923 | 7.155379969 | 34.70849486 | 10.71263748 | 21.43323539 | 14.01267771 | 43.80844569 | 27.96097259 | 43.57896861 | 23.81798944 | 24.94494479 |
| TG(17:2/19:0/21:0) | 0.557089189 | 0.161383962 | 0.807923038 | 0.847874324 | 0.727224738 | 4.973974087 | 18.77057553 | 9.221429727 | 10.45240918 | 6.233538327 | 10.63354108 | 8.790268802 | 15.28565992 | 7.81386007 | 10.10954278 |
| TG(18:1/18:2/22:5) | 0.868748622 | 0.226501687 | 1.066502351 | 0.924810628 | 0.8706529 | 1.725306058 | 4.255986384 | 2.070761711 | 3.735129615 | 3.222282897 | 4.76018027 | 4.325864627 | 4.518064876 | 3.022120912 | 3.758721824 |
| TG(18:1/18:1/22:6) | 1.061723559 | 0.473176555 | 1.085346154 | 0.918586395 | 0.858703535 | 1.994473273 | 5.376409994 | 8.837703273 | 14.01472741 | 2.741142065 | 4.483946684 | 4.2431553 | 4.411194188 | 14.97094386 | 3.674581544 |
| TG(18:3/18:3/22:2) | 0.421095625 | 0.148968967 | 0.406119048 | 0.34583659 | 0.287410611 | 0.743200018 | 2.313730306 | 1.165607352 | 1.696182624 | 1.115606383 | 1.450468371 | 2.226080065 | 3.024394712 | 1.033956329 | 2.279745253 |
| TG(18:2/18:2/22:4) | 0.261616479 | 0.199116967 | 0.699120977 | 0.457573966 | 0.420380727 | 0.980055671 | 2.910602254 | 1.301284344 | 2.267915878 | 1.356855755 | 2.983047626 | 2.736863997 | 4.194655965 | 1.966410148 | 1.940664371 |
| TG(17:1/20:0/20:0) | 0.548518154 | 0.208077314 | 1.069330808 | 0.647818667 | 0.662139191 | 1.958952705 | 10.30635628 | 2.689756389 | 5.495778368 | 3.664159555 | 14.89238005 | 7.595917912 | 12.17132335 | 6.782109986 | 7.627126604 |
| TG(18:0/19:0/20:1) | 0.138323241 | 0.044356636 | 0.208538589 | 0.201722118 | 0.171229059 | 1.156891162 | 3.853208031 | 1.980560937 | 2.2784851 | 1.436134 | 2.38851949 | 2.025456971 | 6.527090754 | 1.734302722 | 2.18568907 |
| TG(18:1/18:1/22:5) | 0.125815607 | 0.039768781 | 0.147209776 | 0.197370199 | 0.117156148 | 0.533073624 | 0.960437718 | 0.833332873 | 1.12896756 | 0.57882894 | 1.095830184 | 1.145464344 | 1.227498168 | 0.717516876 | 1.102931595 |
| TG(18:1/18:1/22:4) | 0.462242436 | 0.155013051 | 2.24889191 | 0.542711563 | 0.480934007 | 1.338342723 | 5.221438075 | 2.157966218 | 3.952142566 | 2.434731788 | 5.533086517 | 5.109114462 | 5.976277448 | 3.603001934 | 4.44371426 |
| TG(18:0/18:0/22:6) | 0.10603382 | 0.041100505 | 0.135498554 | 0.138936293 | 0.122196328 | 0.49336272 | 1.470089756 | 0.729493138 | 0.971443175 | 0.621980076 | 0.889306696 | 0.95047983 | 1.281305196 | 0.776914627 | 0.798539018 |
| TG(18:1/18:1/22:3) | 12.22003403 | 3.113424876 | 13.98024123 | 11.32029381 | 0.63921365 | 21.48570527 | 47.83877284 | 27.10275423 | 48.11970363 | 31.01656525 | 52.53389931 | 54.29497678 | 58.40805141 | 40.69875029 | 47.16954561 |
| TG(17:1/20:4/22:6) | 2.596721025 | 0.380950358 | 6.357606091 | 2.151530838 | 2.004199928 | 6.338355389 | 19.58889878 | 9.602341828 | 18.10965017 | 11.61483236 | 20.13717225 | 22.48582733 | 24.89584385 | 13.07852608 | 15.56807553 |
| TG(18:2/18:2/22:0) | 45.70662286 | 15.4054387 | 72.78941002 | 56.45223245 | 4.875556724 | 206.4847994 | 739.8256677 | 271.8006523 | 476.93552 | 316.3963782 | 804.7402761 | 859.348397 | 790.7150612 | 790.2103306 | 880.1436137 |
| TG(17:1/20:4/22:5) | 4.609904268 | 2.375085013 | 4.890455721 | 4.305558704 | 4.286396867 | 7.468342316 | 11.86235248 | 8.046923282 | 10.99747607 | 9.069416115 | 12.58047054 | 18.12553017 | 11.35384935 | 9.786204792 | 12.02635776 |
| TG(18:1/18:1/22:1) | 28.65607309 | 9.922463952 | 70.90751589 | 35.1544724 | 36.80789047 | 124.1295645 | 437.1001632 | 156.0466217 | 259.5118126 | 171.3745022 | 502.8882512 | 389.2401481 | 459.783006 | 295.6399863 | 309.6865591 |
| TG(18:0/18:2/22:1) | 0.032438976 | 0.011229034 | 0.050129341 | 0.038780397 | 0.043472431 | 0.158205148 | 0.381077903 | 0.280571173 | 0.246562127 | 0.211935526 | 0.519267047 | 0.387539257 | 0.576596086 | 0.16055317 | 0.384367051 |
| TG(17:1/20:3/22:5) | 5.525717539 | 2.312341092 | 5.437714137 | 5.002838398 | 4.658000436 | 9.557282988 | 14.84293395 | 10.34786071 | 14.37881364 | 10.52816497 | 15.53876163 | 15.2602258 | 14.41409154 | 12.58375249 | 13.15921195 |
| TG(18:1/18:1/22:0) | 22.60633064 | 6.033398045 | 36.24636091 | 20.84414595 | 7.649538859 | 75.69127177 | 342.5886296 | 90.71433491 | 164.0802851 | 115.4160579 | 424.8776445 | 227.2101739 | 361.2083823 | 235.398193 | 230.1406877 |
| TG(17:0/20:3/22:5) | 7.105294119 | 1.580725703 | 8.863588163 | 4.258294625 | 4.181165754 | 13.12459985 | 32.91125992 | 12.95800113 | 19.91385351 | 15.16310057 | 24.76987926 | 23.3576652 | 26.7145137 | 19.82916034 | 19.56817904 |
| TG(18:2/19:0/22:6) | 4.303163681 | 0.475090668 | 6.486521877 | 3.069228687 | 2.314413287 | 13.02429901 | 46.92808673 | 25.55958469 | 29.09376006 | 20.83758781 | 45.67214964 | 51.05461344 | 67.47860599 | 25.7947383 | 39.08399363 |
| TG(18:0/18:1/22:0) | 3.772311322 | 1.782327758 | 11.86973745 | 5.581696455 | 6.16257512 | 29.05284224 | 119.8411419 | 37.93113545 | 41.78254997 | 29.76428611 | 169.39041 | 65.46521199 | 122.5256992 | 74.76648888 | 71.68042053 |
| TG(17:0/20:3/22:4) | 6.466122013 | 2.145291218 | 6.237874689 | 4.844856166 | 4.674193546 | 10.60111089 | 28.13600566 | 15.02135985 | 25.11525999 | 17.8861193 | 27.93760343 | 26.13137918 | 28.45806067 | 20.25333401 | 22.56266733 |
| TG(18:0/20:0/20:0) | 0.208304689 | 0.124042627 | 0.583345205 | 0.262873222 | 1.192102523 | 0.885440962 | 5.823236841 | 0.925838255 | 2.042827361 | 1.341082685 | 9.267264647 | 2.968461043 | 5.955651317 | 3.874013371 | 3.42784479 |
| TG(20:4/20:4/20:5) | 9.762243721 | 1.023120802 | 9.542946147 | 5.470500841 | 0.424121219 | 23.16030882 | 168.7423429 | 29.35013607 | 90.26199884 | 51.00872694 | 147.9302025 | 201.2915329 | 192.027918 | 101.149815 | 97.31177482 |
| TG(17:0/20:4/22:2) | 2.84755853 | 0.941857401 | 3.626126473 | 2.718820393 | 3.024763431 | 8.65481521 | 26.69396728 | 11.86075755 | 20.4526751 | 15.24582431 | 25.8666552 | 23.21507772 | 26.25088674 | 12.32696176 | 21.22732781 |
| TG(16:0/22:6/22:6) | 130.6349078 | 24.31516579 | 118.9685402 | 88.68912894 | 72.35379997 | 220.6145569 | 997.3345856 | 327.8025314 | 656.96999 | 376.6098171 | 898.3129384 | 1261.510052 | 1163.494616 | 557.565839 | 707.6119921 |
| TG(16:0/21:0/22:5) | 1.375994859 | 1.437301473 | 5.587117485 | 1.615755535 | 0.018157335 | 4.115024191 | 13.10904888 | 11.97860009 | 10.82964583 | 7.900591883 | 27.5563617 | 11.57081577 | 14.03777072 | 8.966100144 | 20.35353223 |
| TG(18:1/20:4/22:6) | 211.7042775 | 40.15792446 | 224.4186277 | 173.9886133 | 15.74097042 | 496.315749 | 1737.37164 | 928.6036291 | 1218.804089 | 802.5855137 | 2205.439491 | 1570.432758 | 1607.465116 | 990.4765778 | 1294.054791 |
| TG(18:0/19:0/22:4) | 45.45107824 | 19.09826855 | 81.30793404 | 42.20484764 | 37.49096645 | 70.08475644 | 149.5500119 | 134.4163786 | 139.9459448 | 98.95052749 | 254.370808 | 167.63451 | 288.7612768 | 122.3436989 | 140.7922728 |
| TG(16:0/22:5/22:5) | 302.3777535 | 60.37750536 | 267.2327691 | 225.3408444 | 262.9264544 | 329.3162286 | 2207.370796 | 962.8337895 | 1139.035217 | 1176.0983 | 1947.507159 | 1897.9556 | 2067.251852 | 1412.89766 | 1638.242651 |
| TG(20:3/20:3/20:3) | 0.976649593 | 0.080571819 | 1.476331378 | 1.091422853 | 0.65492516 | 10.2828417 | 32.93460088 | 20.68298301 | 29.05658475 | 12.89287707 | 35.38264596 | 26.89758576 | 45.53536169 | 18.55406127 | 25.91343468 |
| TG(17:2/21:0/21:0) | 0.742691376 | 0.279126166 | 1.323463342 | 0.859958071 | 0.840734394 | 2.466715835 | 10.72665575 | 3.287255914 | 6.437703279 | 4.47782912 | 15.12773775 | 8.658061842 | 13.34060027 | 8.119134652 | 8.499621228 |
| TG(16:1/22:2/22:5) | 2.869334587 | 0.458689827 | 4.400981233 | 3.696387088 | 1.019873274 | 19.48070951 | 57.45939734 | 37.79896795 | 50.6372373 | 28.48937831 | 54.52056115 | 45.31522101 | 68.86874764 | 25.829868 | 50.84712147 |
| TG(20:2/20:2/20:3) | 103.9249169 | 22.52382221 | 87.13413825 | 74.9001184 | 77.00475991 | 192.116449 | 734.8792445 | 240.2438221 | 357.3662304 | 424.6015079 | 435.2785206 | 608.8780032 | 456.2798153 | 368.1808976 | 390.5143014 |
| TG(20:2/20:2/20:2) | 58.17623277 | 22.194592 | 85.71634806 | 73.5153601 | 1.037574528 | 213.5119935 | 601.6491634 | 268.2665652 | 450.2260233 | 374.8793677 | 775.2345855 | 455.6337805 | 779.4611182 | 460.4442401 | 488.3484734 |
| TG(18:3/20:2/22:0) | 36.70473768 | 11.35001169 | 63.72267722 | 30.79453008 | 15.63791591 | 105.5119033 | 525.0948151 | 137.2413454 | 340.1076925 | 236.0442395 | 548.7381372 | 264.26198 | 544.1796672 | 385.1111473 | 397.7206449 |
| TG(17:0/22:5/22:5) | 0.978138678 | 0.115543916 | 1.301844727 | 1.09339181 | 0.714568141 | 8.205024254 | 26.26808128 | 22.65304498 | 16.9524529 | 11.20491601 | 26.19752842 | 18.7545087 | 33.67139168 | 12.30867725 | 22.91280261 |
| TG(20:1/20:1/20:1) | 6.794683649 | 2.67469111 | 15.91246259 | 8.74249174 | 2.586565938 | 8.871193644 | 153.8367938 | 41.50340677 | 72.55214353 | 65.63679838 | 193.9945495 | 118.3278637 | 162.035712 | 111.8126813 | 119.2335156 |
| TG(20:0/20:1/20:1) | 3.503952943 | 1.476858494 | 13.29185931 | 4.655845931 | 2.186845986 | 18.93976105 | 97.64691788 | 29.67818622 | 37.45875289 | 41.20084596 | 135.2958187 | 79.87066033 | 133.7687143 | 68.91093948 | 61.63578525 |
| TG(18:3/22:6/22:6) | 1.631519671 | 0.303807167 | 1.794873153 | 2.189773172 | 0.161672873 | 0.989095956 | 28.48065409 | 0.053338589 | 6.947064134 | 3.216187628 | 26.30986568 | 28.59231182 | 31.16274751 | 16.37445044 | 11.52504798 |
| TG(20:0/20:0/20:1) | 1.517618189 | 0.692590474 | 4.148770239 | 2.11675773 | 2.429786971 | 7.588397003 | 21.08748043 | 3.419004865 | 15.80941981 | 11.70601033 | 37.37177594 | 25.08844639 | 22.30767909 | 15.95741407 | 25.96582575 |
| TG(18:2/22:6/22:6) | 31.57385664 | 2.714799805 | 29.99070299 | 15.90848116 | 0.982060567 | 70.60845001 | 487.4891281 | 96.59853455 | 225.2440214 | 145.1072846 | 416.8630379 | 540.3754723 | 509.7972095 | 268.0488064 | 259.8689894 |
| TG(18:2/21:0/22:5) | 28.55002234 | 7.622169066 | 32.04126561 | 26.56231422 | 0.951947231 | 47.77102502 | 108.9359207 | 60.38388152 | 215.2501601 | 70.56090652 | 135.7513617 | 257.5418958 | 118.9011173 | 87.29039416 | 220.6264285 |
| TG(20:0/20:0/20:0) | 0.022057987 | 0.001634539 | 0.023945307 | 0.027926804 | 0.022908741 | 0.108884227 | 0.326568915 | 0.191036317 | 0.202126597 | 0.102200627 | 0.128010615 | 0.154400585 | 0.22650127 | 0.143851381 | 0.185115764 |
| TG(18:1/22:6/22:6) | 85.10190819 | 10.48623408 | 87.74240943 | 59.70714825 | 12.92424795 | 175.8690941 | 750.8872443 | 237.1549896 | 443.1592042 | 328.695898 | 644.4982706 | 722.5021605 | 732.5280625 | 441.3219321 | 489.5339053 |
| TG(18:0/21:0/22:6) | 43.42155922 | 15.52494591 | 49.48279994 | 42.34748377 | 6.884214039 | 73.79351139 | 161.0069813 | 91.01185339 | 199.6785774 | 102.973419 | 152.3110983 | 164.8139135 | 178.1892057 | 120.8706643 | 139.6561453 |
| TG(18:2/22:5/22:5) | 58.21662 | 24.60985523 | 72.70412413 | 106.5033103 | 52.59102511 | 148.3996819 | 388.5114871 | 183.6157078 | 679.7841003 | 220.6498864 | 346.6691148 | 920.0490895 | 387.7006317 | 244.954594 | 291.5564082 |
| TG(18:1/22:5/22:5) | 77.22194374 | 19.85872815 | 99.70370088 | 72.61795718 | 72.80805143 | 187.2499032 | 495.2699186 | 232.7370367 | 371.9306892 | 285.4284294 | 446.7424376 | 430.1267378 | 459.3613496 | 355.785721 | 386.1365862 |
| TG(20:4/20:4/22:2) | 46.51482639 | 16.38386289 | 83.62190011 | 55.61901882 | 71.78307947 | 52.86256341 | 425.376183 | 221.6813701 | 333.6129424 | 216.9710347 | 396.3957312 | 272.966768 | 399.9441495 | 326.7486842 | 356.4949956 |
| TG(18:2/22:4/22:4) | 0.67445129 | 0.052400382 | 1.178260609 | 0.806010798 | 0.255029956 | 8.117631114 | 27.30302367 | 17.24546019 | 20.90215354 | 13.05880446 | 47.58219724 | 22.77697728 | 35.95519704 | 12.42928124 | 21.11009406 |
| TG(18:1/22:4/22:4) | 25.687411 | 9.024236447 | 33.25036325 | 30.94313548 | 29.18627347 | 78.80920072 | 183.2388379 | 88.88704385 | 137.1717847 | 120.2454544 | 162.6256831 | 139.4131385 | 177.5657458 | 138.5654263 | 154.1314166 |
| TG(20:1/20:1/21:0) | 0.180575032 | 0.086466967 | 0.383742993 | 0.2403179 | 0.240540133 | 0.708220077 | 3.346429497 | 0.975272565 | 1.923029701 | 1.288633683 | 5.039030225 | 2.541374426 | 4.14334824 | 2.519365646 | 2.397910027 |
| TG(19:0/20:1/22:1) | 0.024847645 | 0.010402365 | 0.054737817 | 0.03222839 | 0.038956701 | 0.192841151 | 0.594981916 | 0.27407512 | 0.336253918 | 0.219003179 | 0.552604127 | 0.274026763 | 0.528715871 | 0.316560327 | 0.339461732 |
| TG(18:2/22:2/22:4) | 10.98609121 | 4.334211898 | 16.30499212 | 12.60607287 | 0.059677738 | 31.94512157 | 173.7893465 | 36.26624203 | 60.55256431 | 42.83510235 | 157.492408 | 65.69017942 | 81.1373406 | 62.61660186 | 65.89506786 |
| TG(18:0/22:2/22:6) | 1.263548322 | 0.114720908 | 1.137420311 | 0.797201207 | 0.464227248 | 4.87406734 | 14.18417595 | 9.971090555 | 9.813607218 | 7.12638027 | 15.4982753 | 20.50045966 | 19.40077363 | 6.670123525 | 20.23616299 |
| TG(17:1/22:0/22:0) | 0.009670686 | 0.005234887 | 0.021137897 | 0.015116471 | 0.022199438 | 0.047854114 | 0.255282925 | 0.108072756 | 0.105754176 | 0.169512215 | 0.227093958 | 0.225285068 | 0.221625325 | 0.238404636 | 0.246018319 |
| TG(20:2/20:2/22:3) | 11.18132462 | 3.629362533 | 17.80893362 | 15.14939988 | 3.483273064 | 46.21387906 | 125.5105707 | 86.07724858 | 96.15406887 | 67.04421884 | 109.8499936 | 84.64714156 | 86.16200261 | 98.16459527 | 96.37876828 |
| TG(20:1/20:1/22:4) | 15.72010457 | 5.550239214 | 17.45942459 | 14.27641844 | 12.78647044 | 25.35363529 | 61.9830779 | 30.86703604 | 51.075553 | 36.6978989 | 57.34857989 | 58.5538161 | 59.71535552 | 43.83465632 | 50.28874988 |
| TG(20:3/20:3/22:0) | 7.241305311 | 2.403951743 | 14.69417838 | 10.17085421 | 3.978961447 | 40.20798962 | 130.9917471 | 49.82855711 | 92.20409004 | 60.17270567 | 217.7082769 | 85.40703129 | 130.5952886 | 136.5996113 | 98.08246743 |
| TG(19:0/22:6/22:6) | 0.273223471 | 0.173812028 | 0.358928625 | 0.34222248 | 0.380458781 | 0.699129834 | 1.032563872 | 0.664485145 | 0.837756682 | 0.689404829 | 1.285034649 | 0.701379599 | 0.924175391 | 0.787776758 | 0.960624115 |
| TG(20:0/20:4/22:1) | 3.607000461 | 1.405037892 | 8.49943014 | 4.963595391 | 5.529993141 | 20.70176713 | 89.95556501 | 23.75770649 | 41.8283639 | 31.23285935 | 112.6358287 | 52.9842468 | 90.10025027 | 63.55521946 | 58.32857748 |
| TG(20:1/20:2/22:1) | 1.446788432 | 0.5912091 | 3.330152936 | 2.061319948 | 3.642581805 | 13.4323578 | 63.10099821 | 9.121008277 | 5.965766238 | 5.995681269 | 45.15328132 | 36.75824412 | 60.16329974 | 40.93658903 | 27.4843476 |
| TG(20:1/20:1/22:1) | 0.97569141 | 0.423515864 | 3.139723369 | 1.260593289 | 0.471820096 | 4.909402375 | 26.14156621 | 7.777626679 | 10.96907796 | 10.62931242 | 33.31351961 | 19.48733208 | 25.59791208 | 17.69420748 | 15.59113935 |
| TG(20:4/22:6/22:6) | 3.491052552 | 0.306822769 | 3.490250913 | 2.134219746 | 0.210228205 | 8.377327807 | 69.39524684 | 12.6670893 | 31.23153334 | 19.30672453 | 60.20265057 | 80.2252539 | 70.52330527 | 37.3044326 | 36.06221973 |
| TG(20:1/20:1/22:0) | 0.485550191 | 0.322104778 | 1.059988098 | 0.88246235 | 0.359186117 | 2.01041318 | 18.04873523 | 2.844774075 | 4.539236575 | 3.53808441 | 24.83943634 | 6.918820853 | 12.54658511 | 7.717161516 | 7.207026661 |
| TG(20:4/22:5/22:6) | 5.201557695 | 0.700732582 | 6.274719654 | 4.894468061 | 0.974119375 | 18.89324565 | 56.05421742 | 25.02894012 | 48.77006143 | 35.72675571 | 66.09706952 | 60.04006186 | 76.61467953 | 51.51740791 | 53.12252775 |
| TG(20:0/20:0/22:1) | 0.269723356 | 0.128829728 | 0.297136596 | 0.310149699 | 0.389619375 | 1.048997676 | 5.996066858 | 1.288519202 | 2.462555116 | 1.907997083 | 4.628065335 | 3.67626274 | 4.398977659 | 4.315548666 | 3.585837615 |
| TG(20:4/22:5/22:5) | 15.08139436 | 2.288287147 | 18.67714157 | 13.64542638 | 13.35824355 | 43.6542283 | 172.8260589 | 57.15918237 | 111.6829494 | 78.58902354 | 149.5892865 | 147.8632901 | 160.0711112 | 115.2677596 | 117.2944778 |
| TG(20:3/22:5/22:5) | 16.767721 | 4.203203369 | 27.39215961 | 17.72295097 | 4.85090352 | 44.8563936 | 190.4055346 | 53.38540949 | 126.3622857 | 68.34536654 | 114.7061305 | 98.73859561 | 117.4310631 | 91.3348105 | 136.1596823 |
| TG(20:4/22:4/22:4) | 21.07501595 | 5.185170127 | 27.38858409 | 22.6368065 | 4.743700155 | 31.82296044 | 72.35531856 | 53.40135413 | 105.1648191 | 83.27638209 | 130.7043504 | 58.24189316 | 130.733023 | 86.37910679 | 108.7529818 |
| TG(20:3/22:4/22:4) | 9.048645963 | 3.385486457 | 12.12557017 | 11.99702622 | 10.97248364 | 28.80784076 | 65.03386513 | 34.39793105 | 49.28711949 | 36.89340367 | 62.07563442 | 51.98459353 | 56.80999582 | 54.56797516 | 57.31740319 |
| TG(20:5/22:3/22:3) | 0.609074111 | 0.096869765 | 0.88639156 | 0.692036984 | 0.411675268 | 8.744593208 | 11.77510388 | 10.12312656 | 9.742771202 | 11.65358881 | 11.38251436 | 11.28136522 | 13.83483636 | 5.977238296 | 10.89141469 |
| TG(20:0/22:5/22:5) | 7.689599227 | 4.175091393 | 10.58091386 | 9.920353086 | 3.7661756 | 36.59305057 | 54.20947238 | 28.90303416 | 61.99169342 | 35.76969785 | 52.05684156 | 42.74032306 | 50.64605993 | 46.3218978 | 47.86974429 |
| TG(20:2/22:3/22:4) | 3.952759313 | 1.333404017 | 6.310959981 | 5.034943101 | 5.565496303 | 15.13281401 | 38.81702755 | 17.57603764 | 27.8488311 | 21.39131415 | 51.85272603 | 25.7338499 | 33.62376705 | 45.55842421 | 28.84279631 |
| TG(20:2/21:0/22:0) | 0.036221297 | 0.01437961 | 0.04639634 | 0.040703696 | 0.04769288 | 0.14127003 | 0.503057316 | 0.222455161 | 0.327016593 | 0.183411324 | 0.271484118 | 0.278084725 | 0.37777984 | 0.249820089 | 0.287837809 |
| TG(20:1/22:3/22:4) | 2.59387921 | 0.962082012 | 4.00772678 | 3.457598819 | 3.651267919 | 11.30918236 | 28.83242783 | 13.07407264 | 20.74653367 | 17.27904338 | 24.61331876 | 18.03930625 | 26.11931889 | 22.78536043 | 21.94778226 |
| TG(20:2/22:1/22:4) | 2.00789268 | 0.585225643 | 3.33747986 | 2.887189881 | 3.034355637 | 11.05090965 | 35.68226615 | 15.06558934 | 24.48067811 | 17.80170841 | 31.77639885 | 23.50117055 | 31.54173639 | 26.2452584 | 24.98222069 |
| TG(20:2/22:1/22:3) | 1.076396271 | 0.384412576 | 2.328365181 | 1.526721317 | 0.805251232 | 6.844100108 | 25.95108359 | 7.855770662 | 13.31728364 | 10.45989418 | 30.70242064 | 15.73111657 | 25.76501887 | 20.20759351 | 17.49082375 |
| TG(20:0/22:3/22:2) | 0.918832434 | 0.346293903 | 1.415213008 | 1.166104155 | 0.954466898 | 3.40027808 | 14.63448721 | 3.814319408 | 6.363800988 | 5.510140902 | 20.15714857 | 12.50084847 | 22.03913931 | 10.60162674 | 9.118217604 |
| TG(22:6/22:6/22:6) | 1.394304783 | 0.113073344 | 1.083742442 | 0.63135597 | 0.651521541 | 2.088782476 | 19.40958685 | 2.635797331 | 6.832247523 | 4.35159807 | 16.06331125 | 23.59842406 | 20.50397806 | 9.893105101 | 8.253496464 |
| TG(22:5/22:6/22:6) | 2.076234677 | 0.229317118 | 2.127032649 | 1.294897522 | 1.499577227 | 5.507349476 | 42.99174076 | 7.837755011 | 20.0039249 | 10.91159781 | 30.66986152 | 33.46393175 | 32.27721958 | 21.40405739 | 23.9529057 |
| TG(20:1/22:1/22:1) | 0.228521155 | 0.089355881 | 0.405025805 | 0.357945041 | 0.296253079 | 0.91935454 | 4.632361174 | 1.170676185 | 2.889324177 | 1.950761103 | 5.859220275 | 3.62719728 | 5.624658003 | 2.99278724 | 3.182700379 |
| TG(22:4/22:6/22:6) | 3.284401188 | 0.392292153 | 3.746346815 | 2.631897456 | 2.711603353 | 9.393519211 | 46.0441421 | 12.65621201 | 26.25358567 | 17.50839579 | 38.79262634 | 63.8473202 | 46.35566006 | 53.25118579 | 27.8159781 |
| TG(20:0/22:1/22:1) | 0.243770749 | 0.059428774 | 0.302084812 | 0.178026751 | 0.197689252 | 0.564364676 | 2.93457543 | 0.857099754 | 2.270965215 | 1.467491271 | 4.017304335 | 3.144055221 | 4.268790354 | 1.955170123 | 2.219935151 |
| TG(22:5/22:5/22:5) | 4.684639792 | 0.942968684 | 6.173714613 | 5.169296415 | 4.957082255 | 15.12306963 | 49.15762822 | 18.23069308 | 32.08512469 | 24.04390991 | 39.74145185 | 39.46483526 | 42.39416662 | 33.82417292 | 35.76096987 |
| TG(22:1/22:6/22:6) | 1.965070868 | 0.901707299 | 4.568089382 | 2.478749175 | 2.311748036 | 6.5846323 | 24.59211104 | 9.85734556 | 6.788580048 | 8.581721149 | 20.36211407 | 8.888506524 | 10.47868162 | 7.792843643 | 10.85392256 |
| TG(22:4/22:4/22:4) | 1.017037311 | 0.43398453 | 3.885688105 | 1.465096856 | 2.022637257 | 6.43854691 | 4.040531545 | 7.120581166 | 6.599271883 | 9.809217007 | 13.49551367 | 5.724359261 | 8.714094665 | 9.048043665 | 11.47034098 |
| TG(22:3/22:4/22:4) | 1.899087733 | 0.742178547 | 2.663236406 | 2.327010768 | 2.265722236 | 5.551047339 | 13.70486834 | 6.077708987 | 10.02490368 | 11.81014567 | 10.92444417 | 8.419880963 | 11.20868413 | 10.70547293 | 9.540737683 |
| TG(22:0/22:5/22:5) | 1.238667482 | 0.483677037 | 2.115627143 | 2.429818143 | 2.011734809 | 7.752380097 | 19.08777114 | 6.499059288 | 9.649966166 | 11.37170312 | 16.42125605 | 8.8406653 | 15.64417129 | 11.14154785 | 14.34037316 |
| TG(22:1/22:4/22:4) | 1.410883008 | 0.239102665 | 2.13945645 | 0.846697382 | 0.988084822 | 3.521484403 | 9.980923173 | 7.121468701 | 12.41102292 | 5.432462701 | 15.1422484 | 6.599901395 | 9.030640246 | 7.121002701 | 12.45961666 |
| TG(22:2/22:2/22:4) | 0.700361177 | 0.255638156 | 1.307302825 | 0.934785734 | 1.068070867 | 3.880632485 | 12.94472656 | 3.567245758 | 8.378454213 | 5.857994707 | 7.24297328 | 4.322688429 | 11.62833405 | 5.651329924 | 9.337696343 |
| TG(22:1/22:3/22:3) | 0.225667322 | 0.147836235 | 1.057042555 | 0.359251701 | 0.36042355 | 1.731798715 | 5.476571762 | 2.023454924 | 3.373649924 | 2.760651255 | 12.06154319 | 3.208521235 | 5.469812309 | 4.447490196 | 3.742960424 |
